# Exposure to per- and polyfluoroalkyl substances and inflammatory bowel disease: review and meta-analysis

**DOI:** 10.1038/s41370-026-00851-0

**Published:** 2026-03-24

**Authors:** Celina Nicole Phillipson, Scott Michael Bartell

**Affiliations:** 1 Department of Environmental and Occupational Health, Joe C. Wen School of Population & Public Health, University of California, Irvine, CA USA; 2 Department of Statistics, University of California, Irvine, CA USA

**Keywords:** PFAS, Epidemiology, Ulcerative colitis, Crohn’s disease, Risk of bias

## Abstract

**Background:**

Per- and polyfluoroalkyl substances (PFAS) are stable chemicals that are well preserved in the environment and persist in the human body. PFAS have been associated with inflammatory bowel disease (IBD), but studies are varied in their findings.

**Objective:**

The objective of this study was to conduct a review of the literature and meta-analysis evaluating PFAS exposure and IBD.

**Methods:**

PubMed, SCOPUS, Cochrane Library, and the Web of Science were searched for original research articles published in English between 1980 and July 2023 and with human subjects. An adapted risk of bias grading chart was created a priori in order to assess the risks of bias relevant to the studies. For each health outcome, the relative risk point estimates and confidence intervals were used in the synthesis of results.

**Results:**

Six studies were identified and assessed for risk of bias. Using random effects meta-analysis for the four studies reporting odds ratios and confidence intervals for serum PFAS concentrations, we found that the average relative increase in ulcerative colitis per 1 ng/mL increase in measured or modeled serum PFOA for these studies is 7% (95% CI: –4.7%, 20.0%). For other PFAS chemicals, there were only three or fewer studies reporting odds ratios for PFAS and IBD.

**Significance:**

Our results indicate that there is an increased risk of developing ulcerative colitis with increasing serum PFOA; however, the effect is not statistically significant, and has substantial uncertainty. Further research and studies are warranted to enhance the precision of these estimates and clarify the nature of this association.

**Impact:**

This literature review and meta-analysis evaluate the epidemiologic evidence linking PFAS exposure to irritable bowel disease (IBD)—specifically ulcerative colitis and Crohn’s disease. Results were heterogeneous, and meta-analyses yielded null findings across all PFAS-outcome pairs. Risk of bias due to exposure misclassification, reverse causation, and inadequate control sampling limited confidence in the findings. These results highlight the need for future research and rigorously designed studies to evaluate PFAS as environmental risk factors for IBD.

## Introduction

Per- and polyfluoroalkyl substances (PFAS) are a class of extremely stable man-made chemicals that have been used worldwide since the 1940s [1]. PFAS are a family of nearly 5000 chemicals used in industrial applications and consumer products [2]. Many PFAS are extremely stable and resilient to degradation, facilitating their preservation in the environment and long serum half-lives within the human body [3]. The scientific evidence linking PFAS to adverse health outcomes is growing, with environmental epidemiological studies linking reduced birth weight, reproductive and developmental effects, altered immune function, dyslipidemia, and some cancers with PFAS exposure [4–8].

Inflammatory bowel disease (IBD) is a chronic disease characterized by inflammation of tissues in the digestive tract. The two types of IBD are ulcerative colitis—a condition which affects the colon and rectum, and Crohn’s disease—a condition which can affect all parts of the gastrointestinal tract, including the deeper layers of the digestive tract. IBD represents a continuum of diseases, with ulcerative colitis presenting milder symptoms in patients, while patients with Crohn’s disease can experience painful and debilitating symptoms that can lead to life-threatening complications [9]. The underlying cause of IBD is through immunological processes; however, the pathogenesis of the disease is unknown, and presently, no known cure exists [10].

The first report of IBD-related symptoms was recorded in 1793 [11] by Matthew Baille, with the term ulcerative colitis being coined by Samuel Wilks in 1859 [12]. Historically, IBD has been linked to industrialization, and its evolution has been categorized into four stages: Emergence, Acceleration in Incidence, Compounding Prevalence, and Prevalence Equilibrium [13]. In Western Europe and North America, IBD emerged in the 18th and 19th centuries, with full clinical distinction between ulcerative colitis and Crohn’s disease established in 1932 [14]. By the 1950s, Western countries began to experience an acceleration in the incidence of IBD cases, whereas the emergence of IBD in Asian and Latin American countries began in the early 1950s to the late 20th century. Presently, certain Western countries [15] have seen a drop in incidence of new cases; however, because IBD is an incurable, chronic disease with a low mortality rate, the cases of IBD are compounding, shifting stabilizing countries into the Compounding Prevalence stage. The fourth stage, Prevalence Equilibrium, is a hypothetical stage in which the incidence of IBD equals mortality among those with the condition, causing prevalence to stabilize. As the global burden of disease increases rapidly in newly industrialized countries and continues to compound in previously industrialized countries, researching the prevention of IBD is imperative to stemming the global rise of the disease.

PFAS have been associated with the development of IBD, but studies have varied in their findings. In animal studies, PFAS have been found to decrease T-cell-dependent and independent antibody responses (TDAR and TIAR), and decrease disease resistance in host infection studies [16]. PFAS can influence the physical barrier in the intestine by triggering cell apoptosis in intestinal epithelial cells or by creating oxidative stress [17–19]. In epidemiologic studies, some studies have found an association between PFAS exposure and the development of ulcerative colitis and/or Crohn’s disease, but some newer studies have found no association or inconsistent associations pertaining to PFAS exposure. This systematic review seeks to clarify the association between PFAS and IBD in humans.

## Methods

The literature review and meta-analysis were prepared in accordance with Preferred Reporting Items for Systematic Reviews and Meta-Analyses (PRISMA) 2020 statement guidelines, with the completed PRISMA checklist attached in the Supplementary materials (Supplementary Table 1). The protocol was developed and drafted a priori and registered with PROSPERO on April 23, 2023 (CRD42023416782) before developing the risk of bias grading chart, conducting the literature search, extracting data, and synthesizing the results.

### Eligibility criteria

Eligibility criteria were defined using the PECOS (Population, Exposure, Comparator, Outcome, Study design) framework [20].

- Population (P): Human participants of any age. Animal studies were excluded.
- Exposure (E): Studies that measured or modeled exposure to one or more per- and polyfluoroalkyl substances (PFAS). Exposure assessment could include directly measured serum PFAS concentrations or modeled serum levels; studies using proxy indicators of PFAS exposure were also eligible.
- Comparator (C): Studies were included regardless of whether a comparison group was explicitly defined, provided that quantitative associations between differing levels of PFAS exposure and health outcomes were reported. Regression, for example, is a valid technique for comparing health effects at different exposure levels without forming a distinct comparison group.
- Outcome (O): Any health-related outcome relevant to the review question (as specified in the main text). Studies that did not quantify PFAS exposure were evaluated for qualitative summaries but excluded from the meta-analysis of epidemiological effect estimates.
- Study design (S): Original experimental, case-control, cohort, or cross-sectional observational studies published in English. Reviews, meta-analyses, conference abstracts, editorials, letters, and commentaries were excluded.

Studies that duplicated previously published data or did not present original results were excluded.

### Information sources

#### Search strategy

The following databases were searched in July 2023 in order to identify studies to include in the meta-analysis: PubMed, SCOPUS, Cochrane Library, and the Web of Science. Searches were limited to original research articles published in English between 1980 and July 2023 and with human subjects. Keywords and controlled vocabulary were used to describe each outcome. The following search terms were used: (“ulcerative colitis” OR “Crohn’s disease” OR “irritable bowel syndrome” OR “irritable bowel disease” OR “IBD” OR “microscopic colitis” OR “non-specified colitis”) AND (“perfluoroalkyl” OR “polyfluoroalkyl” OR “PFAS” OR “PFOA” OR “PFOS” OR “PFHxS” OR “PFDA” OR “PFNA”). We updated our literature search in October 2025 using the same databases and search terms as the original review.

### Selection process

**Fig. 1:**
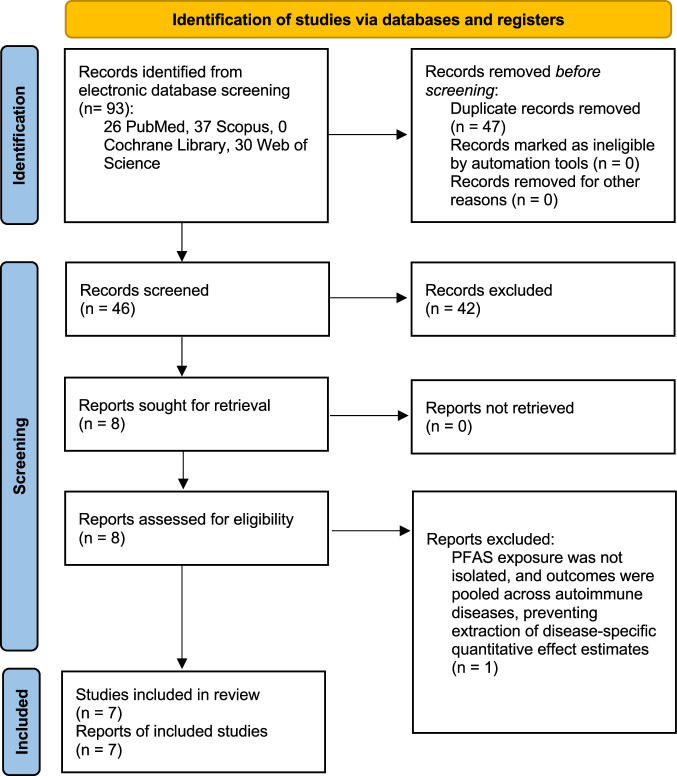
PRISMA flow diagram of study selection process. Flowchart depicting identification, screening, eligibility assessment, and inclusion of studies examining the association between PFAS exposure and inflammatory bowel disease (IBD).

First author, CNP, independently assessed the title and abstract in initial screening. Full-text screening was performed to assess if the inclusion criteria were met by the study. A PRISMA flow diagram was used to record and summarize the screening process (Fig. 1).

### Data collection process

For each study that met the inclusion criteria, the first author, publication year, country, year of method of exposure assessment, outcome assessment, primary exposure pathway, exposure classification, study design, population characteristics, sample size, confounders, and results were collected. Results were collected as risk ratios (which can be approximated by odds ratios) with standard errors (SEs) or 95% confidence intervals. CNP collected the data and verified with the second author, SMB. The eligible outcomes sought were inflammatory bowel disease, ulcerative colitis, and Crohn’s disease.

### Data items

#### Risk of bias assessment

Study quality was assessed by both reviewers using an adapted risk of bias chart (Table 1). The World Health Organization (WHO)—Risk of bias assessment instrument for systematic reviews informing the WHO Global Air Quality Guidelines was selected due to the relevance of the six domain categories in relation to epidemiological studies: confounding, selection bias, exposure assessment, outcome measurement, missing data, and selective reporting. In particular, the WHO —Risk of bias assessment instrument was selected after thorough review and discussion of other similar review tools due to its inclusion of observational studies and particular considerations of validity in environmental epidemiologic studies. While many Risk of Bias tools have been developed in order to increase transparency and reproducibility in systematic reviews, some tools place emphasis on randomized control trials as a gold standard and classify all observational studies as low to moderate quality [21–23]. However, randomized control trials often use limited sample sizes and short follow-up times, which are both inadequate for observing chronic or rare diseases that need larger cohorts and longer observation periods [24]. Furthermore, randomized control trials are most often not possible in environmental epidemiology due to ethical concerns (i.e., study participants can not be randomly assigned to receive toxic exposures). Thus, many epidemiologists argue that placing randomized control trials as the gold standard for occupational and environmental studies can create inappropriate grading guidelines, recommending instead using a grading scale that includes quality of exposure assessment, confounding control, and other sources of bias for observational studies of exposure-response relationships [24]. For this review, we developed a detailed rubric capturing the domains listed in the WHO—Risk of Bias assessment instrument, re-ordered and re-structured to include the following: selection bias, information bias (which was subset into exposure assessment and outcome assessment), bias due to confounding, reporting, and other statistical considerations. The grading system of high, moderate, low, and unclear was retained in order to evaluate the likely magnitude of bias in our tool.

**Table 1:** Criteria for assessing risk of bias of individual studies (adapted from the World Health Organization—Risk of bias assessment instrument for systematic reviews informing WHO Global Air Quality Guidelines, 2020).

Bias Domain	Criteria	Risk of bias
1. Selection Bias	Participants in all exposure levels and with all outcomes had equal opportunity to be in the study, or differential selection was accounted for using appropriate statistical methods (e.g., sampling weights). For case-control studies only, controls were representative of the population that gave rise to the cases, and appropriate statistical methods were used to accommodate oversampling of cases (e.g., logistic regression).	Low
	Participants in all exposure levels (or with all outcomes) did not have equal opportunity to be in the study, but not to the extent that effect estimates were likely to be seriously biased (rationale required, for example, selection was likely to be differential with respect to exposure but not outcomes, or vice versa). For case-control studies only, controls are selected using an established method, but may not be representative of the population that gave rise to the cases (i.e., family-based, hospital- or registry-based for conditions not related to exposure or the outcome).	Moderate
	Participants in all exposure levels (or with all outcomes) did not have equal opportunity to be in the study to the extent that effect estimates were likely to be seriously biased.	High
	Insufficient information to make a judgment.	Unclear
2a. Information bias (exposure assessment)	Based on blood, plasma, or serum PFAS concentrations in samples collected before the health outcome occurred. Based on exposure surrogates/models validated by comparison to measured biomarkers (e.g., correlation coefficient > 0.5, or statistically significant difference in serum PFAS across exposure groups)	Low
	Based on unvalidated but meaningful exposure surrogates/models (e.g., workplace PFAS air concentrations, county-level PFAS water concentrations).	Moderate
	Based on blood, plasma, or serum PFAS concentrations in samples collected after the health outcome occurred.	High
	Insufficient information to make a judgment.	Unclear
2b. Information bias (outcome assessment)	Methods of outcome assessment were comparable across exposure groups.	Low
	Methods of outcome assessment were not comparable across exposure groups; however, evidence supports that outcome detection would not have varied.	Moderate
	Methods of outcome assessment were not comparable across exposure groups.	High
	Insufficient information to make a judgment.	Unclear
3. Bias due to confounding	For studies based on measured blood, plasma, or serum PFAS concentrations, all important confounders were measured with reasonable accuracy and accounted for in analysis: age, sex, race, smoking, and co-exposure among PFAS. For studies based on modeled PFAS concentrations: age, sex, race, and co-exposure among PFAS. Adjustment for a confounder is unnecessary if the confounder is not expected to impact the results (i.e., nearly all the same race in a population, low correlation between PFAS).	Low
	Not all-important confounders were measured with reasonable accuracy and included in the analysis; however, there is evidence that this does not lead to severe confounding, or is not expected to lead to severe confounding due to the relative strength of confounding.	Moderate
	No accounting for important confounders.	High
	Insufficient information to make a judgment.	Unclear
4. Reporting	Complete reporting of all outcomes analyzed, including non-significant results.	Low
	Not all outcomes reported, underreporting of methods or statistical analysis, and not reporting conflicts of interest.	High
	Insufficient information to make a judgment.	Unclear
5. Other Statistical Considerations	Appropriate statistical methods were used.	Low
	Statistical methods used are sensitive to untested assumptions (e.g., complete case analysis for data with a high proportion of missingness, with no evaluation of missingness characteristics).	Moderate
	Inappropriate statistical methods used (e.g., multiple imputation for data with a high proportion of missingness that likely resulted from a “not missing at random” (NMAR) mechanism).	High
	Insufficient information to make a judgment.	Unclear

When developing the tool, special consideration went into each domain category and its specific threats to bias for studies of PFAS exposures and IBD. For selection bias, the study design was regarded to reduce selection bias adequately when participants in all exposure levels and all outcomes had equal opportunity to be in the study, or if authors appropriately adjusted for differential selection by using appropriate statistical methods [25]. For example, in longitudinal studies, selection bias can be corrected by using a generalization of inverse probability weighting [26]. We felt it imperative to include additional considerations for case-control studies in our tool, as cases are intentionally more likely to be sampled than controls, and thus need specific statistical methods to accommodate oversampling of cases (see Table 1 for details). Representative control selection is fraught with challenges, and also needs consideration as a potential source of selection bias when controls are not nested in the same cohort giving rise to the cases. For information bias, both exposure and outcome bias were considered more likely to occur if the methods of assessment were not comparable across groups, which could lead to differential misclassification or measurement errors. The modified instrument included different considerations for potential bias from studies using modeled versus measured PFAS exposure, such as potential bias due to unmeasured physiological confounding. Although measured individual biomarkers have historically been preferred for exposure assessment, more recently, researchers have pointed out that epidemiological studies using modeled exposure based on environmental measurements are less susceptible to physiological confounding and reverse causation, as the more removed a dose estimate is from the individual, the less susceptible it becomes for biases to be impacted by personal factors and physiology [27].

For studies based on measured blood, plasma, or serum, likely confounders such as age, sex, race, smoking, and co-exposure of commonly detected PFAS needed to be measured and incorporated into appropriate statistical models for the study to be considered to have a low risk of bias. Age plays a role in PFAS exposure due to the long half-lives of PFAS, which contribute to cumulative exposure over time, and both menopause and declining kidney function with aging may affect rates of PFAS excretion and bioaccumulation [28, 29]. Age of diagnosis fluctuates in different populations and has changed globally over time, with patterns of diagnosis occurring in peak age ranges typically in adolescence and adulthood, or in bimodal distribution patterns which can include late-onset diagnosis [30–33]. Race has been found to be a determinant of PFAS serum concentrations [34] and is associated with significant differences in IBD incidence [35]. Sex, menopausal status, and hormone use can impact the rate at which someone excretes PFAS, impacting measured exposure status [29]. Studies have shown differences in the pathogenesis of IBD based on sex [36, 37], menopausal hormone use [38, 39], and oral contraceptive use - although the effects differ between ulcerative colitis and Crohn’s disease [40]. Premature menopause was found to be associated with Crohn’s disease [41]; however, the influence of hormones in IBD development and menopause status remains unclear and thus removed from our set of confounders, which needed to be considered low risk of bias [42]. Smoking status has been shown to increase the risk of IBD, with differing effects in ulcerative colitis and Crohn’s disease [43]. PFAS have been found in tobacco and cigarettes, which can impact measured exposure status amongst smokers. Alcohol use was initially considered as a potential confounder; however, multiple studies concluded that alcohol use did not increase the risk of developing IBD and was thus removed from our grading chart [44, 45]. Lastly, co-exposure of PFAS was considered a possible confounder due to previous studies indicating moderate to high correlation amongst different PFAS in the general population [46–48]; however, adjustment was considered unnecessary if PFAS correlations were low in the population studied, as variables that are weakly correlated with exposure require very strong effects on outcomes in order to be important confounders [49]. Due to the lack of a defined etiopathology for IBD, observational studies are also potentially susceptible to spurious findings due to unknown confounding. We view that hypothetical risk of bias as low, even though it is always a possibility in observational studies, considering that important confounders must have moderate to strong relationships with both exposure and disease. A directed acyclic graph can be found in the Supplementary materials that illustrates the pathways between modeled and measured PFAS serum concentrations, confounding variables, and IBD (Supplementary Fig. 1).

In our chart, we included missing data as one of a variety of issues in the category of statistical considerations, and expanded upon possible bias that could arise from missingness as well as bias due to inappropriate statistical methods employed (see Table 1 for details).

#### Synthesis methods

**Fig. 2:**
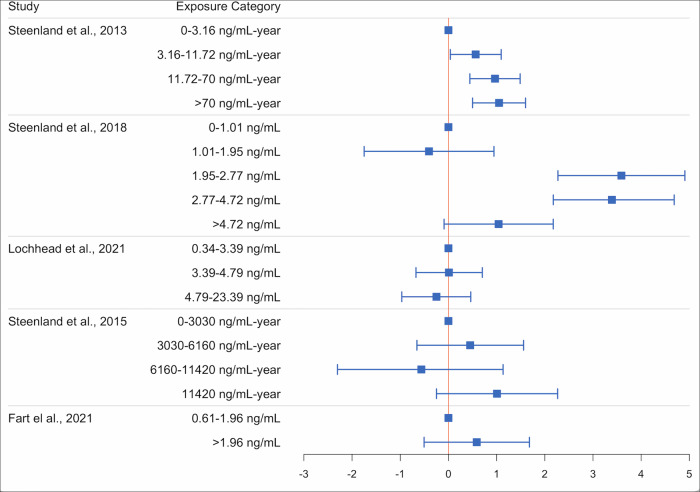
Log rate ratio and 95% confidence interval by study and exposure category for ulcerative colitis and PFOA in human studies.

For each outcome, relative risk (or odds ratio) point estimates and confidence intervals were used in the synthesis of results. The odds ratios (OR) were used in Fig. 2 and Supplementary Fig. 2–9 for each respective PFAS and corresponding health outcome. In all but three studies, Steenland et al. [50], Xu et al. [51], and Agrawal et al. [52], PFAS blood serum measurements were provided in tertiles, quantiles, or quintiles, with cutpoints provided in the original publications. For Steenland et al. [50], Dr. Steenland provided cut points by email at our request. With respect to Xu et al. [51], PFAS were combined and reported as total PFAS in “never”/”ever” exposed categories, or in four time-based categories: “not exposed”, “early”, “mid”, and “late.” Because the time-based categories were based on residential addresses and crude assessments regarding the likely timing of water contamination, with limited interpretability, we chose to use the “never”/“ever” PFAS exposure classification in Supplementary Figs. 3–6. With respect to Agrawal et al. [52], PFAS were investigated as a mixture (without calibration to laboratory standards with known concentrations), and individual PFAS effect estimates could not be extracted from the results. We therefore included this study in our qualitative synthesis but did not incorporate its results into the quantitative meta-analyses, as the combined exposure metric was not comparable to individual PFAS measurements reported in the other studies.

In Fig. 2, Steenland et al. [53] and Steenland et al. [54] used cumulative serum PFOA from birth until the end of follow-up. In order to combine the effect estimates of multiple studies, the results must be reported using the same exposure metrics and outcomes. Thus, we divided the reported cumulative serum PFOA concentrations for each exposure category by 29.9, which is the average age of diagnosis of ulcerative colitis in the United States.

Study results were tabulated in R (R version 4.2.0) under reported PFAS chemical and outcome of interest - either ulcerative colitis or Crohn’s disease (see R code in Supplementary Materials). Only one study, Xu et al. [51], reported effect estimates for subtypes of IBD other than ulcerative colitis or Crohn’s disease; however, because it was the only study to do so, we were unable to perform analysis for their additional category of “other or non-specified IBD.” Three of the seven studies only assessed the relationship of PFOA to ulcerative colitis, and the remaining four studies included results for other PFAS chemicals. In one of the four studies to include other PFAS chemicals, Xu et al. [51], PFHxS and PFOS were found to be elevated in serum measurements amongst 3500 participants six months past the cessation of contamination; however, because the study did not measure or report PFAS in serum from the IBD registry-based study participants, or distinguish between different PFAS chemicals in the water supply, we extracted the same effect estimate for both PFHxS and PFOS for our forest plots (Supplementary Figs. 3–6) but chose exclude the odds ratio in our formal meta-analysis.

For Steenland et al. [50], some analyses included the year of sampling; however, the logistic regression results needed for the meta-analysis published in the original manuscript did not adjust for the year of sampling. This introduces risk of bias due to confounding, as the year of sampling can be a predictor of PFAS serum levels. In order to address this, we reached out to the authors who provided new results that adjusted for the year of sampling. The provided results were used in our figures and meta-analysis, while the original results were assessed for the risk of bias grading chart.

For Fart et al. [55], published results were reported in fold rate ratios; however, through communication with senior author Ida Schoultz, we were able to receive raw data, which included pair number, age at diagnosis, diagnosis year, sampling year, smoking status, and PFAS serum concentration in ng/mL. Age was only provided with respect to age at diagnosis and thus was missing for controls and omitted from our model. Similarly, smoking status was only reported for cases and thus was also omitted from our model. Lastly, the sampling year had no overlap between cases and controls, with controls taken a median of 6 years after cases. As we expect PFAS serum levels to drop over time, we decided to exclude the year of sampling in our model. After categorizing PFAS serum concentrations into two groups based on the median, a conditional logistic regression analysis (clogit) was performed, adjusting for paired strata.

#### Meta-analysis methods

In order to perform meta-analysis, first an average log rate ratio per ng/mL of PFAS was estimated for each study and each PFAS chemical, using inverse-variance weighted regression to combine results across exposure categories within each study [56]. ORs for Fart et al. [55] were calculated via conditional regression analysis using PFAS serum concentrations as a continuous variable and accounting for pair matching, instead of inverse-variance weighted regression, in order to obtain a more precise assessment of how changes in PFAS levels are associated with ulcerative colitis and Crohn’s disease.

We also obtained formal meta-analysis estimates combining the log rate ratios across all studies for each PFAS chemical. Meta-analysis is a statistical technique used to combine effect estimates from multiple studies to derive a single, unified effect estimate and confidence interval that represents the entire body of evidence while weighing the results of each study according to the precision of its effect estimates [57]. We obtained results from two common types of meta-analysis: fixed effects and random effects.

#### Ethical approval

This manuscript is a review article and does not involve a research protocol requiring approval by the relevant institutional review board or ethics committee.

## Results

### Study selection

Our search produced 93 records identified from four databases—PubMed (*n* = 26), SCOPUS (*n* = 37), Cochrane Library (*n* = 0), and Web of Science (*n* = 30). Among these, 47 records were duplicates and removed prior to screening. After screening titles and abstracts, 42 records were removed before record retrieval. Eight records were assessed for eligibility, of which seven met the inclusion criteria and were included in the meta-analysis (Fig. 1).

#### Study characteristics and risk of bias assessment

All studies were assessed and graded based on the risk of bias grading chart (Table 2). Two of the studies investigated PFOA exposure in a highly exposed population in the Mid-Ohio Valley, pursuant to a lawsuit settlement in which the C8 Science Panel was charged to determine whether PFOA was linked to any human disease. The first study listed in our table, [53], included a community cohort of participants who lived or worked in any of the six PFOA-contaminated water districts and participated in a baseline survey. Historical exposure was reconstructed based on PFOA release amounts from the DuPont plant, wind patterns, river flow, groundwater flow, drinking water use, the residential address history provided by each participant, and a pharmacokinetic model; exposure reconstruction was validated with PFOA serum measurements taken in 2005–2006. Medical history was collected during two rounds of interviews and confirmed through medical documentation, with only confirmed medical cases included in the analysis. Because the community cohort was large with high participation rates (>80%), and the exposure and outcome measurements were comparable across all exposure groups, the study was graded to have low risk of bias in all categories. Although other PFAS chemicals were not included in the model, correlations between PFAS were found to be low with respect to PFOA and other PFAS chemicals in the study population [58]. Because of this, adjustment for co-exposure was deemed unnecessary as it was unlikely to impact the results.

**Table 2:** Studies on exposure to PFAS and IBD.

		Study Characteristic	Bias Rating	Reasoning
Study	Bias Domain			
Steenland et al. [53]: Highly exposed community cohort study	Selection Bias	Community cohort—persons who lived or worked in any of six PFOA-contaminated water districts and participated in the C8HP baseline survey in 2005–2006. The combined resident and worker population was 32,254.	Low	Participants of all exposure groups and outcomes had equal opportunity to participate in the study; participation rates were very high (~49% of the total community).
	Information Bias Exposure Assessment	Historical exposure reconstruction with yearly serum PFOA estimates based on the amount of PFOA released from the DuPont plant, wind patterns, river flow, groundwater flow, drinking water use, the residential address history provided by each participant, and a pharmacokinetic model. Estimates of annual serum PFOA levels for workers in different jobs were developed and combined with estimated annual serum levels from residential exposure. Model-based exposure predictions were validated by comparison to serum PFOA measurements in 2005-2006 for community cohort members (*r* = 0.67).	Low	Exposure reconstruction, validated by comparison to measured serum PFOA concentrations.
	Information Bias Outcome Assessment	Participants provided medical history via phone (63%) or online (37%), focusing on chronic disease. Surveys included demographic information and medical history. For participants who died after completing round one of the survey, the next of kin was surveyed (4% of the cohort had next of kin). If a chronic disease was reported, participants were asked about the date of diagnosis and consented to review medical documents. Professional medical abstractors obtained records documenting the disease in question from medical providers by mail or in person. Able to find medical records for 92% of those who consented.	Low	Methods were comparable across exposure groups, and outcome(s) were validated by medical records.
	Bias due to confounding	Included in analysis: age, sex, race and ethnicity (white, non-Hispanic or other), BMI, education, smoking, and alcohol consumption.	Low	All confounders included. High exposure to PFOA only in water; low correlation amongst measured serum PFAS.
	Reporting	Exposure-outcome analysis was hypothesis-driven, from a list of prespecified health conditions. Only medically verified outcomes are used in the analysis. Two types of sensitivity analysis: qualifying time analysis—eliminated prior non-exposed before cohort eligibility (loss of 10–15% of cases depending on outcome). Above background analysis—associations with cumulative exposures above background levels only.	Low	Complete reporting of all health outcomes analyzed.
	Statistical	Associations between PFOA exposure and each outcome were conducted using separate survival analyses (Cox regression) with age as the time variable. For each outcome, follow-up ended at the time of last interview, time of disease occurrence, or at the time of death (whichever occurred earliest), with validated cases only. Estimated associations with cumulative exposure to PFOA, which was calculated by summing estimated yearly concentration during follow-up and modeled as a time-dependent variable in Cox Regression. Also considered cumulative with a lag of ten years. Conducted analysis by quartile of cumulative exposure, with cut points derived for lagged and unlagged exposures. Rate ratios were estimated for each second, third, and fourth quartile relative to the first quartile (referent group).	Low	Appropriate statistical methods were used.
Steenland et al. [54] Worker cohort study	Selection Bias	Included 3713 workers, a subset of two prior studies conducted by the C8 Science Panel, an occupational mortality study, and a combined community/worker study of disease incidence.	Low	Workers were contacted as a part of a community study, allowing participants of all exposure levels and outcome opportunities to have an equal opportunity to be in the study.
	Information Bias Exposure Assessment	Serum PFOA was measured in past and present residents of the six contaminated water districts. For the mortality study, estimated PFOA serum levels over time for these workers were considered by considering occupational and residential exposure (via contaminated drinking water, using the same exposure reconstruction and pharmacokinetic model as in Steenland, 2013, described above). A job exposure matrix (JEM) was created for occupational exposure, with five job categories created based on exposure. Serum samples were matched to the job category to estimate yearly serum levels in each category by using a general linear model, taking correlation of repeated measures into account. Occupational serum estimates implicitly incorporate residential exposure, based on observed serum levels in workers over time. Yearly serum estimates from the occupational exposure model were used for the years when people worked at the plant if these were higher than residential estimates; if they were lower, the residential (community) estimates were used.	Low	Based on serum PFAS concentrations in samples collected before the health outcome occurred, and surrogates/models validated by comparison to measured biomarkers (correlation coefficient > 0.5).
	Information Bias Outcome Assessment	Collected data for a number of chronic disease outcomes through two rounds of interviews— conducted in 2008 and 2011—and retrieved medical records for validation. Potential healthy worker effects are mitigated by questionnaire administration to all former workers, not just current workers.	Low	Methods of outcome assessment are similar across all exposure groups.
	Bias due to confounding	Models included gender, race (Caucasian/non-Caucasian), education, body mass index, smoking status, and alcohol consumption. Models were stratified by year of birth for a 5-year period to control for potential confounding.	Low	All relevant confounders included in the analysis. High exposure to PFOA only in occupation and water; low correlation amongst measured serum PFAS.
	Reporting	Analyses were restricted to outcomes with 20 or more cases and validated cases. The proportional hazards assumption was tested via an interaction term between time (age) and log cumulative exposure, which was not significant in any of the disease-specific analyses.	Low	Reported cases included in analysis, as well as cases not included due to missing interviews or incomplete residential or work history.
	Statistical	Cox regression models with age as the time scale, and time-varying exposure and covariates, to analyze data for the worker cohort. The principal exposure metric was cumulative exposure, with or without a 10-year lag period. Cumulative exposure was calculated as the sum of yearly exposure estimates from birth through any given year.	Low	Appropriate statistical methods were used.
Steenland et al. [50]: General population case-control study	Selection Bias	Cases were recruited from Emory University Hospital on Emory Campus who volunteered for research. Controls were non-diseased friends and family of cases in a previous IBD study, who were asked to enroll in a research study of African-Americans regarding the genetics of inflammatory bowel disease. In reporting of results, CD cases were combined with controls, which could bias results to the null. Healthy controls were slightly older than patients with IBD and had their blood taken later; however, age and sampling year were controlled for in the analysis.	High	Controls were not friends and family of the cases in the study, and thus were not representative of the population that gave rise to the cases.
	Information Bias Exposure Assessment	Stored serum samples were available at the time of the study; however, samples were taken after diagnosis.	High	Blood samples collected after diagnosis of IBD; high potential for physiological effects/reverse causation.
	Information Bias Outcome Assessment	Standard criteria used to establish the diagnosis of UC or CD. Comprehensive demographic, clinical, laboratory, and serologic values were obtained either at the time of enrollment or by retrospective chart review.	Low	Methods of outcome assessment are similar across exposure groups.
	Bias due to confounding	Measured and included in analysis: age, gender, and race (white/non-white, Hispanic/Asians considered non-white). While there are substantial differences between race groups outside of white versus non/white (NHANES), race was controlled for in the analysis and is not a likely threat to validity. UC and CD serum samples were taken from 2004 to 2013 (mean 2008) and 2005 to 2012 (mean 2008). For controls, sampling years ranged from 2010 to 2013 (mean 2011). Year of sampling can be a predictor of PFAS serum levels, as PFAS levels are expected to be lower for later sampling years based on NHANES data. Thus, controls are expected to have lower levels of PFAS concentrations due to a lag in sampling. Furthermore, some analysis adjusted for the year of sampling; however, the logistic results published in the original manuscript did not adjust for the year of sampling. The final analysis did not adjust for co-exposure among the PFAS chemicals, which are expected to be highly correlated among background levels.	Moderate	Year of sampling and co-exposure among PFAS was not adjusted for in the original analysis.
	Reporting	Results reported with and without 4 outliers. Final analysis included 4 outliers.	Low	Complete reporting of all outcomes analyzed.
	Statistical	Standard linear regression with PFOA and other PFAS as outcome variable, logistic regression with UC/CD as outcome.	Low	Appropriate statistical methods were used
Xu et al. [51]: Highly exposed community cohort study	Selection Bias	Cohort of residents in Ronneby municipality, at least one year between 1980 and 2013 (*n* = 63,074).	Low	Information obtained from residential addresses and registries, participants of all exposure levels had an equal opportunity to participate.
	Information Bias Exposure Assessment	Based on yearly residence addresses (from Statistics Sweden) and an archive of water supply. Residents with private water wells are considered unexposed to measurements. The exact date of contamination of the water supply was unknown; the authors set 1985 as the start of exposure. Crude exposure assessment using a dichotomous exposure variable (never/ever living at an address with contaminated water after 1985), then a four-category time-based variable (not exposed 1980–1984, early 1985–1994, mid 1995–2004, late 2005–2013) was included in the model.	Moderate	Exposure categories based on residence addresses, with waterworks data available from 2013 and on.
	Information Bias Outcome Assessment	Health outcomes were collected from two national registries: 1. The Swedish Patient National Register, which included hospital in-patient visits since 1987 and both in-patient and out-patient from public and private visits from 2001 and on. 2. cause-of-death register.	Low	Methods of outcome assessment were comparable across exposure groups.
	Bias due to confounding	Models included gender, age, and education level. Additional adjustment for education level as a proxy for socioeconomic status. Age was included in the models, but large age categories were used in the analysis (10 years). Race was not included in the model; however, the population is mostly homogenous. Smoking/alcohol status not needed due to modeled (not measured) exposure. In the analysis, PFAS were combined and presented as total PFAS in “never”/”ever” exposed to PFAS in drinking water based on residential history; however, PFHxS and PFOS were found to be elevated in serum measurements. Analysis did not adjust for the co-exposure of PFHxS and PFOS.	High	No ability to see the individual risk ratio of PFHxS and PFOS. Large age categories may lead to residual confounding.
	Reporting	Sensitivity analysis performed using gender, in-patient versus out-patient registries, age, and education level. All outcomes reported.	Low	Complete reporting of all outcomes analyzed, including non-significant results
	Statistical	Cox proportional hazards model, calendar year as time. Analyses for in-patient only, in-patient and out-patient, subjects below 45, above 45, and education level (proxy for SES).	Low	Appropriate statistical methods were used.
Lochhead et al. [65]: General population cohort study	Selection Bias	Nurses’ Health Study (NHS) is a nationwide prospective cohort study initiated in 1976, in a cohort of nurses aged 30-35 years at baseline (*n* = 121,701). NHS II was initiated in 1989, with a cohort of nurses aged 25-42 years at baseline (*n* = 116,686). Follow-up exceeded 85%. Cases were matched to two control individuals free of IBD, and matched on cohort, age, month of blood collection, fasting status, menopausal status, and menopausal hormone therapy used at the time of blood collection.	Low	Participants of all exposure groups were able to be included in the study.
	Information Bias Exposure Assessment	73 cases of CD and 80 cases of UC from NHS I and NHS II, obtained from blood samples collected before diagnosis or indexing.	Low	Based on plasma samples collected before the health outcome occurred.
	Information Bias Outcome Assessment	Follow-up questionnaires have been administered every two years since baseline with open-ended questions to report UC/CD. From 1982 onward, the diagnosis of UC was included in the questionnaire and in 1992 for CD. Medical records granted access for 80% of cases.	Low	Outcomes verified by medical records and two gastroenterologists.
	Bias due to confounding	Included in analysis: body weight, menopausal status, menopausal hormone use, smoking status, leisure-time physical activity, oral contraceptive use, parity, state of residence, and co-exposure of PFAS. No significant differences between cases and controls for matching factors: age, menopausal status, menopausal hormone use, and time interval between blood collection and diagnosis. Basic model controlled for cohort, age, month of blood draw, menopausal status, menopausal hormone therapy, and PFAS. Multivariable model adjusted for potential confounding by BMI, smoking status, OC use, parity, physical activity, and region of residence.	Low	Important confounders were included and measured with reasonable accuracy.
	Reporting	For a small portion of cases (CD *n* = 3, UC *n* = 1), plasma was only available from one of the two matched controls. Plasma concentrations lower than LOD were replaced by LOD divided by 2 (applied to only a single control participant).	Low	Outcomes and serum were reported and analyzed.
	Statistical	OR and CI computed using conditional logistic regression models, stratified on match.	Low	Appropriate statistical methods were used.
Fart et al. [55]: General population case-control study	Selection Bias	Cases were recruited at Örebro University Hospital, Örebro and the Karolinska University Hospital, Stockholm. Healthy blood donors without any history of gastrointestinal disease were recruited from Örebro University as controls. Healthy controls were slightly younger than patients with IBD; however, age and sampling year were controlled for in the analysis.	High	Controls may not be representative of the population that gave rise to the cases.
	Information Bias Exposure Assessment	Samples from IBD patients were taken from an outpatient IBD clinic of Örebro University Hospital. Samples were obtained at a median of 7 to 8 years after diagnosis. Control serum samples were taken a few years later than those of IBD patients; however, the year of sampling was controlled for in the analysis.	High	Blood samples collected after diagnosis of IBD; high potential for physiological effects/reverse causation.
	Information Bias Outcome Assessment	Samples from IBD patients were obtained from a previous cohort. Diagnosis of IBD based on clinical, endoscopic, radiologic, and histologic criteria. Clinical data were recorded by a treating physician.	Low	Methods comparable across groups.
	Bias due to confounding	Age (+/– 5 years), sex, and for cases (UC and CD) only - disease duration (+/– 5 years). Year of sampling measured and included in fold rate ratio analysis, but not the final analysis used in meta-analysis. Year of sampling can be a predictor of PFAS serum levels, as PFAS levels are expected to be lower for later sampling years based on NHANES data. Thus, controls are expected to have lower levels of PFAS concentrations due to a lag in sampling. Smoking status was only provided for cases and omitted from the meta-analysis.	High	Not all relevant confounders were included in the analysis.
	Reporting	The majority of PFAS are detected and reported in serum. Values below LOD were replaced by LOD divided by two. Bile acid levels could not be ascertained for one CD sample.	Low	Complete reporting, including non-significant results.
	Statistical	General linear model fitted to investigate associations between PFAS concentration and UC or CD vs controls, presented as fold change ratio.	Low	Appropriate statistical methods were used.
Agrawal et al. [52]: Nested case control of military personnel	Selection Bias	Nested case control platform study of subjects with incident CD and UC and healthy control subjects (*n* = 75). Data crosslinked with the Department of Defense Serum Repository. Incident cases selected based on ≥2 medical encounters with a diagnosis, and available serum at the time of diagnosis ( ± 1 year). Controls required to have no medical encounter with evidence of IBD, rheumatoid arthritis, celiac disease, or colon cancer. Cases and controls were frequency-matched based on age ( + 1 year), sex, and timing of specimen.	Low	Controls representative of the population that gave rise to the cases.
	Information Bias Exposure Assessment	Serum samples were obtained from the Department of Defense Serum Repository at four time points (1–10 years) prior to IBD diagnosis. 300 serum samples from 75 military service members at 4 different time points.	Low	Blood samples were collected before the diagnosis of IBD.
	Information Bias Outcome Assessment	Ambulatory and inpatient medical encounter data, diagnoses identified between 1998 and within the Defense Medical Surveillance System, based on the International Classification of Diseases-Ninth Revision code for CD and UC.	Low	Methods comparable across groups.
	Bias due to confounding	Age, sex, and race-matched controls. PFAS chemicals are assessed as mixtures.	Low	All relevant confounders were included in the analysis.
	Reporting	Quality control subjects included in the pooled sample. Results reported for weighted quantile sum and individual regression analyses. No missing data in covariates, exposures, and outcomes.	Low	Complete reporting, including non-significant results.
	Statistical	Weighted quantile sum regression to estimate the overall mixture association between the PFAS chemical mixture and disease outcomes. For sensitivity, we analyzed random sunset variants of weighted quantile sum from the total set to assess intercorrelation between exposures and summarized overall exposure to the mixture. Individual regression analyses are reported in the Supplementary materials.	Low	Appropriate statistical methods were used.

The second C8 Science panel study, [54] included 3713 workers as a subset from two prior cohort studies, one an occupational mortality study [59] and the other a combined worker and community cohort study [60] following exposure to PFOA. The original worker cohort included 6026 employees who had worked at least 1 day between 1948 and 2002 at the DuPont West Virginia plant, 5791 of whom were included in the mortality study. The combined worker and community study included analysis of retrospective exposure estimates for 3713 participants who underwent two rounds of interviews in which information regarding demographics, smoking, residential history (all residences since birth), medical history, and reproductive history was collected. For reported outcomes, medical records were retrieved to validate self-reported IBD diagnoses for participants who granted consent. In order to estimate occupational exposure, a job exposure matrix was created, which included five job categories based on PFOA exposure. Serum samples were matched to the job category to estimate yearly serum levels in each category by using a general linear model, taking correlation of repeated measures into account. Occupational serum estimates incorporated residential exposure based on observed serum concentrations in workers, as follows. The environmental fate transport model as described in Steenland et al. [53] was used in order to estimate serum PFOA concentrations from residential exposure. Yearly serum PFOA concentration estimates from the occupational exposure model were used for the years when people worked at the plant if these were higher than residential estimates; if they were lower, the residential (community) estimates were used. Models included adjustment for race (Caucasian/non-Caucasian), education, body mass index, smoking status, and alcohol consumption, and were stratified by year of birth for a 5-year period in order to control for potential confounding. Analyses were restricted to outcomes with 20 or more cases with validated cases in Cox Regression models. Age was used as the time scale, with the principal exposure metric being cumulative exposure reported with or without a 10-year lag period. Models controlled for sex, race, education, and BMI as well as time-varying smoking and alcohol consumption. Because the worker cohort was large with high participation rates, the exposure and outcome measurements were comparable across all groups, and based on a model with a Spearman correlation of 0.67 between measured and modeled serum PFOA concentrations, this study was marked to have low risk of bias for all bias domains. Occupational cohorts can be especially prone to selection bias via the healthy worker effect [25]. The healthy worker effect can lead to selection bias as jobs may require able-bodied people, or can lend itself to self-selection as healthy workers are more likely to remain in the workforce (i.e., do not quit or get fired due to chronic diseases) [61]. However, because follow-up occurred outside of the workplace as part of the previous community cohort, this study was considered to have low risk of selection bias, as the study was not follow up was not limited to time under employment, but also included information collected through post-employment residential surveys.

The third study included for analysis, [50], was a case-control study conducted at Engleston Hospital on the Emory University Campus. Cases were recruited from 1999 to 2012 among patients who volunteered to participate in research. Controls were non-diseased friends and family of cases in a previous case-control study of African-Americans regarding the genetics of inflammatory bowel disease [62]. Since controls were not friends and family of the cases in the Steenland et al. [50] study, they were not necessarily representative of the population that gave rise to the cases. For example, as noted by the authors, the cases included were mostly white, whereas the controls were mostly non-white. Because controls were not selected using an established method, and there was evidence of stark demographic differences between cases and controls, we marked this study to have high risk of selection bias. At the time of enrollment or by retrospective chart review, comprehensive demographic, clinical, laboratory, and serologic values were obtained and stored for future studies. Serum PFOA, PFOS, PFHxS, and PFNA concentrations were measured in both the cases and controls; however, the mean sampling year differed between the cases and controls (2008 vs 2011). Year of sampling was included in exploratory regression models with log PFOA as the outcome variable; however, it was not included in the final logistic regression for ulcerative colitis or Crohn’s disease vs controls in relation to PFOA. Per nationally representative data from NHANES, year of sampling is a strong predictor of PFAS serum concentrations, with steadily declining PFOA, PFOS, and PFHxS serum concentrations over the last few decades [63]. As such, controls that are sampled years later than cases are expected to have lower levels of PFAS concentrations regardless of case status, simply due to the lag in sampling time. We marked this study as having moderate risk of bias due to confounding due to this issue.

In addition, blood samples for cases were collected after diagnosis, with cases providing blood serum for storage after confirmation of diagnosis. Because of this, this study was marked to have a high risk of bias for exposure assessment due to the high potential for physiological effects or reverse causation from IBD affecting PFAS elimination and serum concentrations [64].

Although the year of sampling differed between cases and controls without statistical adjustment in the published logistic regression model of Steenland et al. [50], we requested and received new results from Dr. Steenland that included adjustment for year of sampling. Thus, the results that were used in our meta-analysis included adjustment for the year of sampling, mitigating the risk of confounding bias compared to the previously published effect estimates.

The logistic regression model also included three age categories, gender, and race (white/non-white, Hispanic/Asian considered non-white). It should be noted that there are differences between race groups outside of white versus non/white (NHANES) in PFAS serum concentrations; however, race was controlled for in the analysis and was deemed not a likely threat to validity. The final analysis did not adjust for co-exposure among the PFAS chemicals, which are expected to be highly correlated among background levels found in a general population cohort. Thus, the study was marked to have low risk of bias for information assessment - outcome assessment, reporting, and statistical analysis and moderate risk of bias due to potential confounding for year of sampling and PFAS co-exposures, and high risk of bias due to information assessment - exposure assessment due to potential physiological confounding from IBD-induced changes in PFAS excretion.

The fourth study, included [51], was a retrospective cohort study based in a community exposed to PFAS-contaminated municipal drinking water. Similar to the two C8 study papers, the cohort consisted of participants who had resided in the contaminated area, and exposure assessment was based on historical residential addresses and waterworks supply information. Because of the large cohort and methodology of collecting highly complete residential addresses from Statistics Sweden, selection bias was marked as a low risk of bias. For exposure assessment, exposure was based on reported residential addresses, and modeled water exposure was based on archived water supply data. The waterworks data were only available starting in 1993, producing some uncertainty regarding the timing and duration of exposure. The hazard ratio was reported in either two classes, “Never” or “Ever” exposed via contaminated drinking water, or four classes labeled “Never”, “Early”, “Mid”, and “Late” describing the timing of residence in an area with contaminated drinking water. Because exposure was crudely modeled and no quantitative exposure assignments were provided for the four class exposures, the two class exposures of “Never” or “Ever” were used for the study summaries in Supplementary Figs. 3–5, and the results from this study were excluded from the meta-analysis. Because of the lack of quantification and uncertainties regarding the timing and magnitude of exposures, this study was considered to have a moderate risk of information bias for exposure assessment. Health outcomes were collected from two national registries: The Swedish Patient National Register, which included hospital in-patient visits since 1987 and both in-patient and out-patient from public and private visits from 2001 and on, and the cause-of-death register. As the method of outcome assessment was comparable across exposure groups, the study was regarded as having a low risk of information bias in outcome assessment. The Cox proportional hazards models included gender, age, and education level as proxies for socioeconomic status. Race was not included in the model; however, the population is mostly racially homogenous, obviating the need for adjustment. In the analysis, total PFAS was presented as “Never”/”Ever” exposure categories based on residential history. Biomonitoring samples taken six months after the cessation of contamination, but not reported in the paper, showed elevated levels of PFHxS and PFOS in more than 3500 participants. Analysis did not adjust for the co-exposure of PFHxS, PFOS, and PFOA despite high correlations found in the Ronneby population. Furthermore, the age categories used in analysis spanned 14 to 24 years, which could lead to residual confounding, as age is a strong a priori confounder. As such, the study was regarded to have high risk of bias due to confounding. Sensitivity analysis was performed using gender, in-patient versus out-patient registries, age, and education level, for all outcomes reported via restriction, and was thus deemed to have low risk of reporting bias.

The fifth study, [65], was based on the Nurses’ Health Study, a nationwide prospective cohort using parallel cohorts established in 1976 and 1989 of female registered nurses who provided health and lifestyle information every two years via follow-up questionnaires. For both cohorts, follow-up exceeded 85% and exceeded 100,0000 participants in each cohort. IBD diagnosis was self-reported in an open-ended section in the questionnaires until 1982, when ulcerative colitis was added as a specific question, and until 1992 for Crohn’s disease. If reported, a supplementary questionnaire was provided and medical records access requested; 80% of whom consented to medical record review. Body weight, menopausal status, menopausal hormone use, smoking status, leisure-time physical activity, oral contraceptive use, parity, state of residence, and co-exposure of PFAS were measured and controlled for in order to account for possible confounders. Each case was matched to two control individuals who were free of IBD at the time of cases’ diagnosis and were matched on cohort, age, month of blood collection, fasting status, menopausal status, and menopausal hormone therapy use at the time of blood collection. No significant differences between the cases and controls were found for matching factors: age, menopausal status, menopausal hormone use, and time interval between blood collection and diagnosis. The multivariable logistic regression model adjusted for potential confounding by BMI, smoking status, OC use, parity, physical activity, region of residence, and co-exposure of PFAS. For this study, risk of bias was marked as low for all bias domains due to the large participation rates, ubiquity of records provided and accessed between all exposure and outcome groups, all appropriate confounders measured and accounted for, and appropriate statistical analysis used.

The sixth study included in our analysis, [55], was a case-control study in which serum was collected from patients with late-onset IBD (55 years of age and older). Cases were recruited from a previous study [66] at Örebro University Hospital, Örebro and the Karolinska University Hospital, Stockholm. Healthy blood donors without any history of gastrointestinal disease were recruited from Örebro University as controls. Controls were slightly younger on average than patients with IBD; however, age was accounted for in the analysis and thus not deemed a threat to selection bias. Serum samples from IBD patients were taken from an outpatient clinic at Örebro University Hospital at a median of 7 to 8 years after diagnosis. Serum samples from healthy control participants were taken a few years later than those from IBD patients. Year of sampling was controlled for in the analysis, which minimized the risk of information bias in exposure assessment; however, because the samples were collected after the diagnosis occurred, the study was marked to have a high risk of bias for exposure assessment due to a high potential for physiological effects or reverse causation. Analysis included PFAS as the only covariate analyzed in the model; thus, the study was considered to have a high risk of bias due to confounding. For all other domains, the risk of bias was graded to be low.

The final paper in our analysis, [52], was a nested case-control study of military personnel with Crohn’s disease or ulcerative colitis. Serum samples were obtained from the Department of Defense Serum Repository at four different time points (1–10 years) prior to diagnosis. 25 cases for each health outcome (Crohn’s disease and ulcerative colitis, respectively) were selected based on prior diagnosis of IBD and available serum measurements at time of diagnosis (within 1 year of diagnosis) using ambulatory and inpatient medical data within the Defense Medical Surveillance System. Controls were required to have no medical encounter with evidence of IBD, rheumatoid arthritis, celiac disease, or colon cancer and were frequency-matched based on age ( + 1 year), sex, and timing of serum specimen. PFAS were assessed as a mixture, with models adjusting for age at diagnosis and different batches of chemical exposure analysis. This study found a substantial increase in the odds of CD and UC among those with higher serum PFAS mixture levels up to 10 years prior to diagnosis. For this study, risk of bias was marked as low for all bias domains due to multiple serum measurements taken prior to diagnosis, outcome ascertainment relying on consistent medical encounter data using ICD-9 codes, confounding was appropriately addressed by matching and by assessing PFAS as chemical mixtures, and all relevant covariates were included in the analyses. Reporting and statistical methods were robust, with complete data, inclusion of non-significant results, and use of weighted quantile sum regression to assess overall mixture effects while accounting for intercorrelations among exposures.

All outcomes were analyzed and reported, and results were presented as a fold rate change analyzed via a general linear model to investigate associations between PFAS concentration and ulcerative colitis or Crohn’s disease vs controls. As mentioned in our methods section, raw data were provided to us by the senior author, Dr. Schoultz, for the calculation of comparable relative risk for use in our meta-analysis.

#### Results of synthesis

The studies were arranged in descending order from the highest number of cases to lowest in our summary figures (Fig. 2 and Supplementary Figs. 2–10). Figure 2 and Supplementary Figs. 1–9 show the point estimates and confidence intervals for the log (base e) rate ratios in each study and exposure category. A log rate ratio of 0 indicates that the incidence of ulcerative colitis or Crohn’s disease does not differ between that exposure group and the referent group, while a positive value indicates that ulcerative colitis or Crohn’s disease incidence is more common in that exposure group than the referent group. PFOA and ulcerative colitis had the most studies included (*n* = 5), followed by PFOA and Crohn’s disease, PFHxS and ulcerative colitis, PFHxS and Crohn’s disease, PFOS and ulcerative colitis, and PFOS and Crohn’s disease, each of which included three studies (Supplementary Figs. 2–10). There were three studies each on PFNA and PFDA with both outcomes (Supplementary Figs. 2–10), Agrawal et al. [52], not pictured in figures due to inability to extract point estimates.

The estimated effect of PFOA on ulcerative colitis is heterogenous amongst studies, and does not exhibit a clear dose-response gradient, with increased exposure consistently exhibiting increased risk amongst the study populations. The studies included in Fig. 2 are heterogeneous in exposure assessment methods, with three studies Steenland et al. [50], Lochhead et al. 65, and Fart et al. [55] based on serum measurements, and the others using occupational exposure reconstruction and/or residential histories in relation to PFAS drinking water contamination. Of the three, only Lochhead et al. 2021 used stored serum samples collected prior to IBD diagnosis and found no increased risk of developing ulcerative colitis with increased exposure to PFOA. Steenland et al. [50] and Fart et al. [55] reported positive associations, but were rated to have a high risk of bias due to serum measurements taken after diagnosis of disease and in different time periods for cases versus controls. The other two studies included in Fig. 2, Steenland et al. [53] and Steenland et al. [54], were based on modeled serum measurements in highly exposed communities. While modeled serum based on residential and/or occupational exposure histories can have significant measurement error or exposure misclassification, usually causing a bias towards null associations, methodologists have pointed out the advantage of modeled exposure from external exposure measurement, having resistance to physiological biases such as physiological confounding and reverse causation [27]. Of the five studies included for PFOA and ulcerative colitis, Steenland et al. [53] is the only study that fit a continuous dose-response model. Control sampling methods also differed across the case-control studies, with only Lochhead et al. 2021 using nested case-control, guaranteeing that the controls were sampled from the same study base giving rise to the cases. The heterogeneity of study methodology and potential bias arising from sampling taken after diagnosis could explain the heterogeneity in results displayed in Fig. 2.

The studies listed in Tables 3 and 4 used various measures of association, such as risk ratios, odds ratios, and hazard ratios and were considered comparative measures used in the meta-analysis. The meta-analysis results displayed in Tables 3 and 4 show no significant increase or decrease in risk of developing ulcerative colitis or Crohn’s disease for any PFAS chemical; however, only two to five papers were included in each meta-analysis, suggesting further studies are needed to lower the margin of error. All studies were considered to have a low risk of reporting error due to detailed and apparently complete reporting of methods and results; however, two studies had a high risk of bias due to confounding, and one study had a moderate risk of bias due to confounding. Two studies were graded to have a high risk of selection bias and a high risk of exposure assessment information bias, and one study was marked to have a moderate risk of exposure assessment information bias. Because of this, the confidence in the body of evidence was regarded as low, due to inconsistencies across a small number of studies, many of which have a moderate to high risk of bias.

**Table 3:** Rate ratio for ulcerative colitis (95% CI) per 1 ng/mL increase in serum.

PFAS	# of studies	Fixed-Effects Meta-Analysis	Random Effects Meta-Analysis
PFOA	5	1.00 (1.00, 1.00)	1.07 (0.95, 1.20)
PFHxS	2	0.98 (0.97, 0.99)	0.98 (0.97, 0.99)
PFOS	2	0.99 (0.98, 1.00)	1.04 (0.92, 1.19)
PFNA	2	1.01 (0.98, 1.05)	1.21 (0.64, 2.28)
PFDA	2	1.17 (0.72, 1.90)	1.28 (0.58, 2.81)

**Table 4:** Rate ratio for Crohn’s disease (95% CI) per 1 ng/mL increase in serum.

PFAS	# of studies	Fixed-Effects Meta-Analysis	Random Effects Meta-Analysis
PFOA	3	0.99 (0.96, 1.01)	0.99 (0.96, 1.01)
PFHxS	2	0.99 (0.98, 1.00)	0.99 (0.98, 1.00)
PFOS	2	0.99 (0.99, 1.00)	0.99 (0.99, 1.00)
PFNA	2	0.74 (0.53, 1.02)	0.74 (0.53, 1.02)
PFDA	2	0.23 (0.10, 0.52)	0.23 (0.10, 0.52)

## Discussion

This review summarizes the findings of existing literature investigating ulcerative colitis and Crohn’s disease with exposure to PFOA, PFHxS, PFOS, PFNA, and PFDA. Although significant associations were reported in some of the individual studies, meta-analysis using both fixed effects and random effects showed no clear increase or decrease in risk with increased exposure to any of the five PFAS chemicals included in the analysis. Ulcerative colitis and PFOA had the largest number of studies included in our meta-analysis, with five studies, while all other meta-analyses included just two or three studies. The small number of studies and lack of consistent results indicate a need for further studies. Three of the five studies for PFOA and ulcerative colitis were based on exposure models that estimate serum concentrations over time based on occupational exposure and/or residential exposure to contaminated water. In both, a one-compartment pharmacokinetic model that uses a half-life of 3.5 years for PFOA based on previous historical exposure [67]; however, a recent meta-analysis found a half-life estimate of 2.73 years for PFOA [68], suggesting some overestimation of exposure in Steenland et al. [53] and Steenland et al. [54]. In both papers, PFOA was found to be positively associated with the risk of developing ulcerative colitis; had these studies used the shorter half-life, the effect estimates for PFOA would have likely been larger than reported (i.e., smaller amounts of estimated exposure producing the same observed increase in health effects).

Only two of the studies reported mutually adjusting for frequently detected PFAS, which could result in bias due to confounding by co-exposures, as PFAS concentrations in serum are often correlated [69]. In all three Steenland studies, only PFOA was assessed, and in the studies that did measure other PFAS chemicals, adjustment for mixtures of co-exposures was not conducted for each individual PFAS chemical. In particular, Lochhead et al. 2021, Fart et al. [55], Agrawal et al. [52] were the only studies to measure PFDA and PFNA, highlighting a gap in existing literature for short-chain PFAS chemicals. Agrawal et al. [52] in particular found positive associations between PFNA and PFDA as part of a PFAS serum mixture with respect to both Crohn’s disease and ulcerative colitis, which could influence our meta-analysis results if we were able to extract the PFAS-specific point estimates [52].

With respect to Fart et al. [55] results, we also chose not to adjust for co-exposure to other PFAS when running the conditional regression analyses to input the reported ORs into our meta-analysis, given the small sample size of 20 cases and 20 controls per health outcome. Even in large datasets, adjusting for co-exposure to other highly correlated PFAS can lead to bias amplification due to residual confounding, as a result of being measured with some degree of error and arising from a shared source [70, 71]. Thus, even if studies did not mutually adjust for co-exposure of PFAS, they were not regarded as having an inherently high risk of bias, as there are trade-offs and limitations to analyzing PFAS chemicals as mixtures, rather than using one-at-a-time exposure analyses. In particular, because the studies included in the meta-analysis ranged from only 20 to 151 cases, mutually adjusting for all measured PFAS chemicals along with confounders would often violate the typical statistical guideline of having at least 10 to 20 observations for each predictor in a regression model [72].

Two studies, Xu et al. [51] and Fart et al. 2020, included toxicology results that measured PFAS in serum and fecal biomarkers or bile acids, shedding light on possible mechanisms for IBD development after PFAS exposure. Although the etiology of IBD remains unknown, a limitation of this review and meta-analysis is the exclusion of mechanistic results that investigate imbalances in the gut microbiome, intestinal barrier function, and bile acid metabolism. As more research is done regarding PFAS and possible biological and genetic impacts, future reviews could expand to include biomarkers and potential immune responses such as T-cell dependent and independent antibody responses (TDAR and TIAR), and decreased disease resistance in host infection studies, which can inform the biological plausibility of observed epidemiological associations.

The findings from this review and meta-analysis have significant implications for practice, policy, and future research for PFAS and its potential as an environmental trigger for IBD. While the findings of this review were inconclusive, healthcare providers should be aware of the potential links between PFAS exposure and IBD. This awareness is crucial for patient history, particularly in regions with known PFAS contamination, such as contaminated water sources or occupational exposures. While policymakers have begun to regulate and set enforceable limits, it remains critical to study short-chain PFAS and potential health effects as they have replaced long-chain PFAS in many countries since the 2000s. For example, while the U.S. Environmental Protection Agency announced its final National Primary Drinking Water Regulation (NPDWR) for six PFAS—PFOA, PFOS, PFHxS, PFNA, HFPO-DA, and mixtures containing two or more PFAS— notably, PFDA has no enforceable federal limits in drinking water. PFDA remains underregulated and understudied in human populations, despite being linked to decreased antibody response to vaccines, hyperactivity, developmental delays, and breast cancer [73, 74].

A key limitation of this meta-analysis is that we combined effect estimates from studies conducted in both general populations and populations with high PFAS exposure, such as occupational cohorts or communities exposed to contaminated drinking water. This approach assumes a linear dose-response relationship, in which associations at low and high exposures are proportional; however, if the slope of the dose-response differs between low- and high-exposure groups, combining these populations could either dilute or exaggerate the overall effect estimates.

Future studies should aim to include larger sample sizes that are sufficient to mutually adjust for co-exposure to various PFAS, which will provide more robust and reliable results by addressing potential confounding biases and identifying whether particular PFAS chemicals are responsible for IBD induction. For case-control studies, serum samples should ideally be collected at the same time between cases and controls. Nested case-control studies using stored blood samples are especially advantageous for minimizing the risk of exposure assessment bias, as demonstrated by Lochhead et al. 65 and Agrawal et al. [52]. Research should focus on a wider demographic to understand how different populations are affected by PFAS exposure, particularly marginalized groups who may have higher exposure levels or barriers to healthcare access. In particular, studies should aim to include countries in Asia, Africa, South Africa and Latin America that have seen a steady increase in incidence since the 1990s but have been historically underrepresented in IBD clinical trials [75–77].

This review highlights the complexity of studying exposure to PFAS and its association to IBD and the need for comprehensive, well-designed studies to clarify potential environmental triggers for autoimmune diseases.

## Supplementary information

Supplementary information

## Data Availability

All data generated or analyzed during this study are included in this published article under Supplementary materials.
