# Supplementary material for: Exposure to per- and polyfluoroalkyl substances and inflammatory bowel disease: review and meta-analysis
Source: J Expo Sci Environ Epidemiol. 2026 Mar 24;36(4):640–55. doi: 10.1038/s41370-026-00851-0 (PMC13331747; doi:10.1038/s41370-026-00851-0)
Supplement: Supplementary file 1 — Supplementary information [file 41370_2026_851_MOESM1_ESM.docx]

**Supplemental Material**

**Exposure to per- and polyfluoroalkyl substances and inflammatory bowel disease: A Systematic Review and Meta-Analysis**

Celina Nicole Phillipson and Scott Michael Bartell

**Table of Contents**

**Table S1.** PRISMA Checklist

**Figure S1.** Directed Acyclic Graph of modeled and measured serum PFAS and IBD

**Figure S2**. Log rate ratios for human studies of PFOA and Crohn’s disease

**Figure S3.** Log rate ratios for human studies of PFHxS and ulcerative colitis

**Figure S4.** Log rate ratios for human studies of PFHxS and Crohn’s disease

**Figure S5.** Log rate ratios for human studies of PFOS and ulcerative colitis

**Figure S6.** Log rate ratios for human studies of PFOS and Crohn’s disease

**Figure S7.** Log rate ratios for human studies of PFNA and ulcerative colitis

**Figure S8.**  Log rate ratios for human studies of PFNA and Crohn’s disease

**Figure S9.** Log rate ratios for human studies of PFDA and ulcerative colitis

**Figure S10.** Log rate ratios for human studies of PFDA and Crohn’s disease

**R Code**

**Table S1.** PRISMA Checklist

| **Section and Topic** | **Item #** | **Checklist item** | **Location where item is reported** |
| --- | --- | --- | --- |
| **TITLE** | | |  |
| Title | 1 | Identify the report as a systematic review. | Title page, Lines 10-11 |
| **ABSTRACT** | | |  |
| Abstract | 2 | See the PRISMA 2020 for Abstracts checklist. | Page 2, Lines 32-57 |
| **INTRODUCTION** | | |  |
| Rationale | 3 | Describe the rationale for the review in the context of existing knowledge. | Page 3-4, Lines 61-97 |
| Objectives | 4 | Provide an explicit statement of the objective(s) or question(s) the review addresses. | Page 4, Line 97 |
| **METHODS** | | |  |
| Eligibility criteria | 5 | Specify the inclusion and exclusion criteria for the review and how studies were grouped for the syntheses. | Page 5, Lines 108-125 |
| Information sources | 6 | Specify all databases, registers, websites, organisations, reference lists and other sources searched or consulted to identify studies. Specify the date when each source was last searched or consulted. | Page 5, Lines 116-122 |
| Search strategy | 7 | Present the full search strategies for all databases, registers and websites, including any filters and limits used. | Page 5, Line 131 |
| Selection process | 8 | Specify the methods used to decide whether a study met the inclusion criteria of the review, including how many reviewers screened each record and each report retrieved, whether they worked independently, and if applicable, details of automation tools used in the process. | Page 6, 155-157 |
| Data collection process | 9 | Specify the methods used to collect data from reports, including how many reviewers collected data from each report, whether they worked independently, any processes for obtaining or confirming data from study investigators, and if applicable, details of automation tools used in the process. | Page 6, Lines 160-165 |
| Data items | 10a | List and define all outcomes for which data were sought. Specify whether all results that were compatible with each outcome domain in each study were sought (e.g. for all measures, time points, analyses), and if not, the methods used to decide which results to collect. | Page 5, Lines 119-121 |
|  | 10b | List and define all other variables for which data were sought (e.g. participant and intervention characteristics, funding sources). Describe any assumptions made about any missing or unclear information. | Page 5, Lines 110-114 |
| Study risk of bias assessment | 11 | Specify the methods used to assess risk of bias in the included studies, including details of the tool(s) used, how many reviewers assessed each study and whether they worked independently, and if applicable, details of automation tools used in the process. | Page 6-9, Lines 170- 246 |
| Effect measures | 12 | Specify for each outcome the effect measure(s) (e.g. risk ratio, mean difference) used in the synthesis or presentation of results. | Page 9, Lines 249-252 |
| Synthesis methods | 13a | Describe the processes used to decide which studies were eligible for each synthesis (e.g. tabulating the study intervention characteristics and comparing against the planned groups for each synthesis (item #5)). | Page 9-11, Lines 249-305 |
|  | 13b | Describe any methods required to prepare the data for presentation or synthesis, such as handling of missing summary statistics, or data conversions. | Page 9-11, Lines 223-264 |
|  | 13c | Describe any methods used to tabulate or visually display results of individual studies and syntheses. | Page 9-11, Lines 223-264 |
|  | 13d | Describe any methods used to synthesize results and provide a rationale for the choice(s). If meta-analysis was performed, describe the model(s), method(s) to identify the presence and extent of statistical heterogeneity, and software package(s) used. | Page 11-12, Lines 308-319 |
|  | 13e | Describe any methods used to explore possible causes of heterogeneity among study results (e.g. subgroup analysis, meta-regression). | Page 11-12, Lines 308-319 |
|  | 13f | Describe any sensitivity analyses conducted to assess robustness of the synthesized results. | Supplemental material: R code |
| Reporting bias assessment | 14 | Describe any methods used to assess risk of bias due to missing results in a synthesis (arising from reporting biases). | Page 11, Lines 291-305 |
| Certainty assessment | 15 | Describe any methods used to assess certainty (or confidence) in the body of evidence for an outcome. | Pages 7-, Lines 187-192 |
| **RESULTS** | | |  |
| Study selection | 16a | Describe the results of the search and selection process, from the number of records identified in the search to the number of studies included in the review, ideally using a flow diagram. | Page 12, Lines 324-328, Figure 1 |
|  | 16b | Cite studies that might appear to meet the inclusion criteria, but which were excluded, and explain why they were excluded. | Figure 1 |
| Study characteristics | 17 | Cite each included study and present its characteristics. | Pages 12-18, Lines 331-513, Table 2 |
| Risk of bias in studies | 18 | Present assessments of risk of bias for each included study. | Pages 12-18, Lines 331-513, Table 2 |
| Results of individual studies | 19 | For all outcomes, present, for each study: (a) summary statistics for each group (where appropriate) and (b) an effect estimate and its precision (e.g. confidence/credible interval), ideally using structured tables or plots. | Figure 2, Supplemental Figures 2-10 |
| Results of syntheses | 20a | For each synthesis, briefly summarise the characteristics and risk of bias among contributing studies. | Table 2 |
|  | 20b | Present results of all statistical syntheses conducted. If meta-analysis was done, present for each the summary estimate and its precision (e.g. confidence/credible interval) and measures of statistical heterogeneity. If comparing groups, describe the direction of the effect. | Pages 19-21, Lines 516-563 |
|  | 20c | Present results of all investigations of possible causes of heterogeneity among study results. | Pages 19-21, Lines 527-563 |
|  | 20d | Present results of all sensitivity analyses conducted to assess the robustness of the synthesized results. | Supplemental material: R code |
| Reporting biases | 21 | Present assessments of risk of bias due to missing results (arising from reporting biases) for each synthesis assessed. | Table 2 |
| Certainty of evidence | 22 | Present assessments of certainty (or confidence) in the body of evidence for each outcome assessed. | Pages 21, Lines 561-563 |
| **DISCUSSION** | | |  |
| Discussion | 23a | Provide a general interpretation of the results in the context of other evidence. | Page 20, Lines 497-514 |
|  | 23b | Discuss any limitations of the evidence included in the review. | Pages 20 - 22, Lines 497-558 |
|  | 23c | Discuss any limitations of the review processes used. | Page 21-22, Lines 525-545 |
|  | 23d | Discuss implications of the results for practice, policy, and future research. | Pages 22 - 23, Lines 546-570 |
| **OTHER INFORMATION** | | |  |
| Registration and protocol | 24a | Provide registration information for the review, including register name and registration number, or state that the review was not registered. | Page 4, Lines 100-105 |
|  | 24b | Indicate where the review protocol can be accessed, or state that a protocol was not prepared. | Page 4, Lines 103-105 |
|  | 24c | Describe and explain any amendments to information provided at registration or in the protocol. | n/a |
| Support | 25 | Describe sources of financial or non-financial support for the review, and the role of the funders or sponsors in the review. | Page 31, Lines 897-901 |
| Competing interests | 26 | Declare any competing interests of review authors. | Page 31, Lines 906-909 |
| Availability of data, code and other materials | 27 | Report which of the following are publicly available and where they can be found: template data collection forms; data extracted from included studies; data used for all analyses; analytic code; any other materials used in the review. | References, Pages 24-30, Lines 659-878, Supplemental Materials : R Code |

*From:*  Page MJ, McKenzie JE, Bossuyt PM, Boutron I, Hoffmann TC, Mulrow CD, et al. The PRISMA 2020 statement: an updated guideline for reporting systematic reviews. BMJ 2021;372:n71. doi: 10.1136/bmj.n71. This work is licensed under CC BY 4.0. To view a copy of this license, visit <https://creativecommons.org/licenses/by/4.0/>

**Figure S1.** Directed Acyclic Graph of modeled and measured serum PFAS and IBD


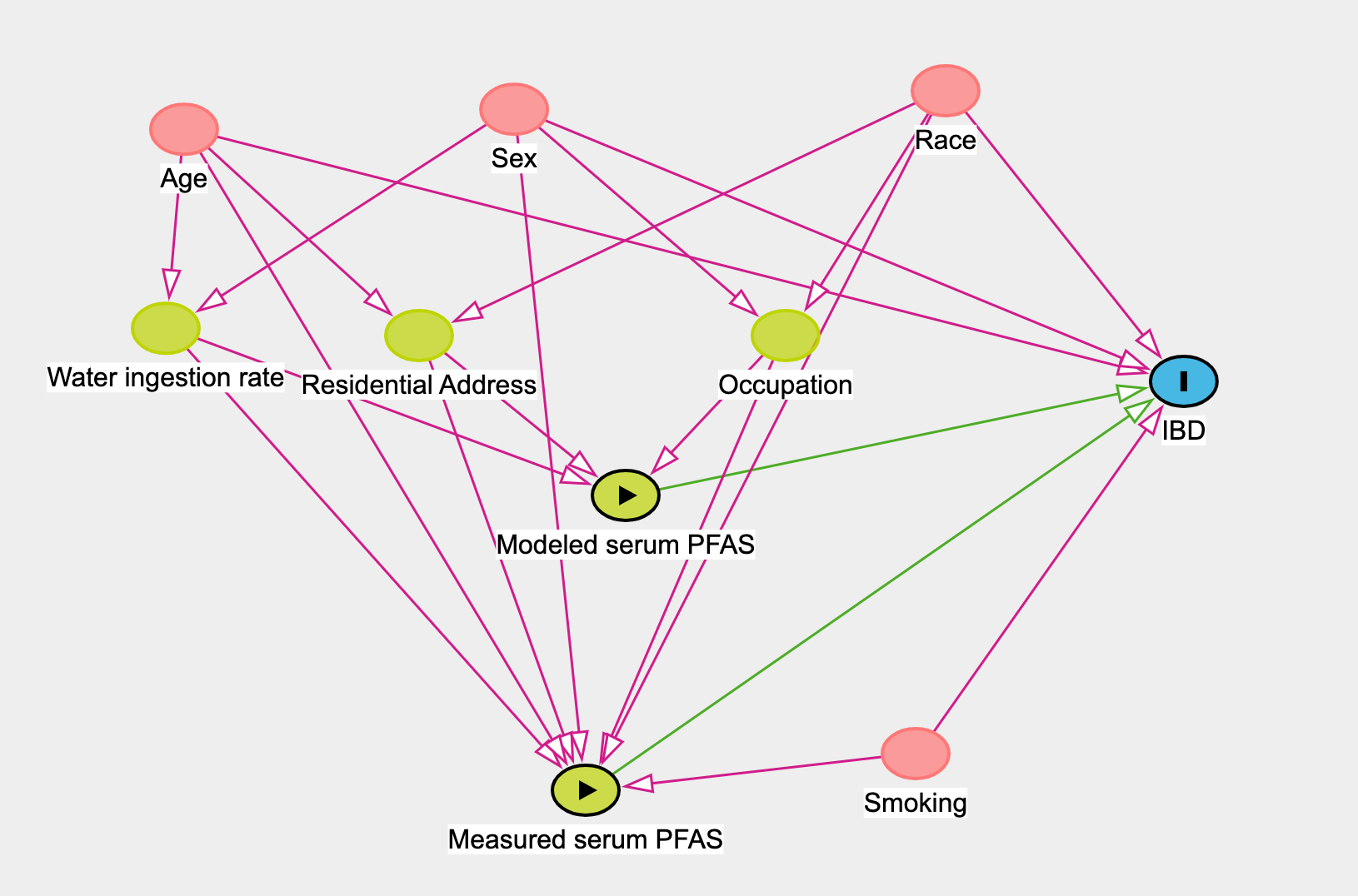


**Figure S2**. Log rate ratios for human studies of PFOA and Crohn’s disease


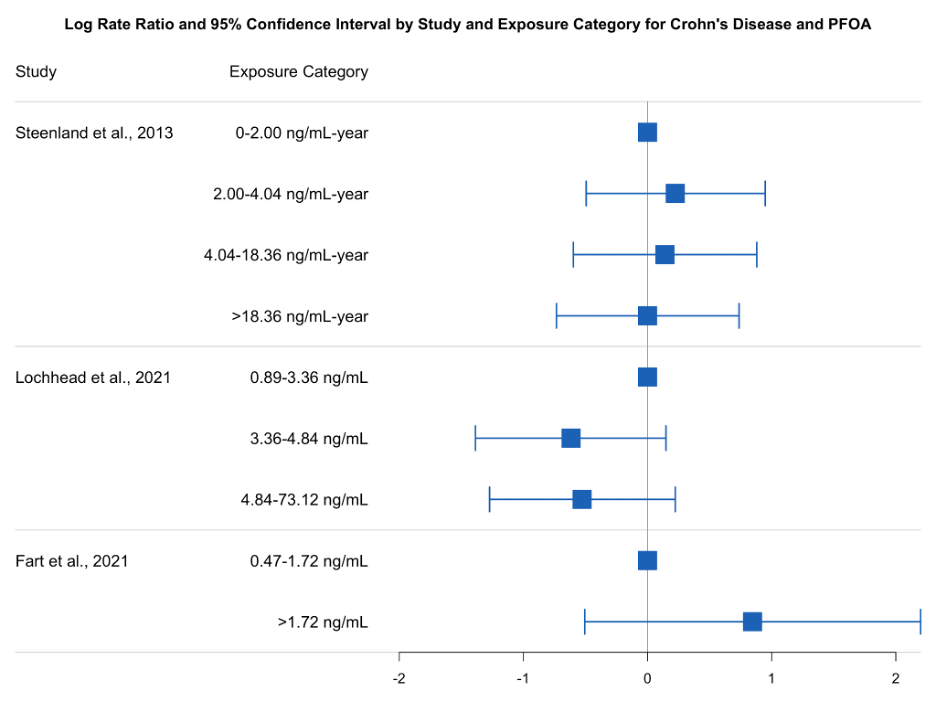


**Figure S3.** Log rate ratios for human studies of PFHxS and ulcerative colitis

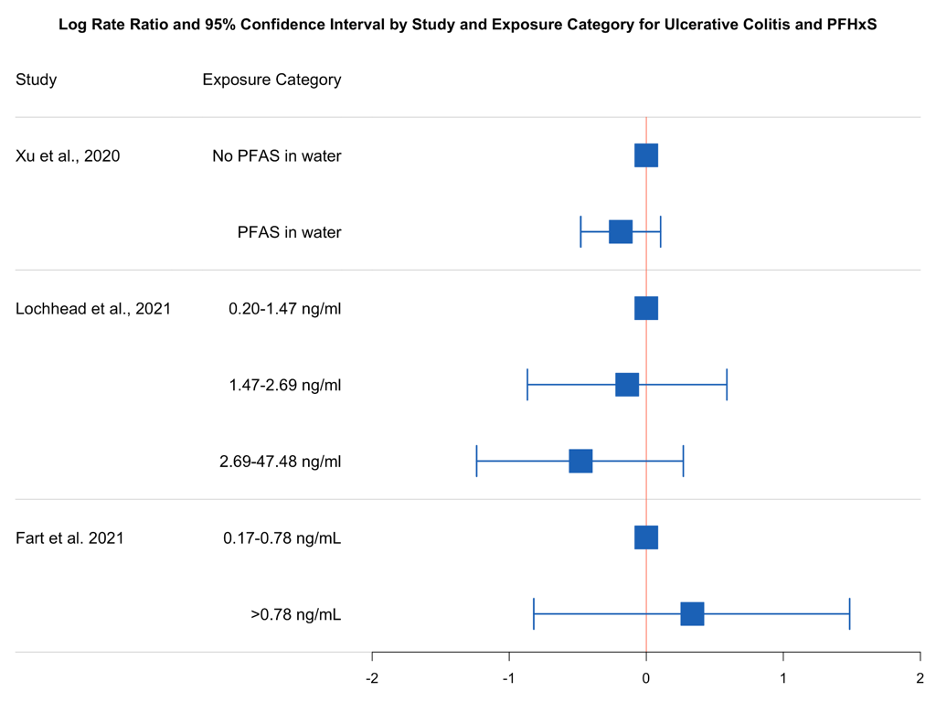


**Figure S4.** Log rate ratios for human studies of PFHxS and Crohn’s disease


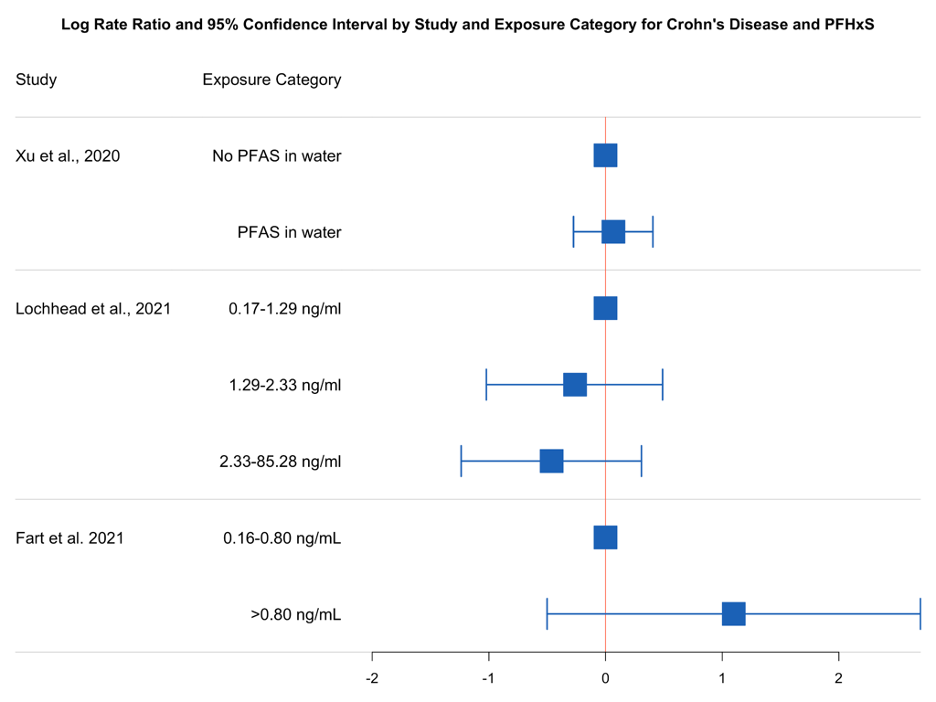


**Figure S5.** Log rate ratios for human studies of PFOS and ulcerative colitis


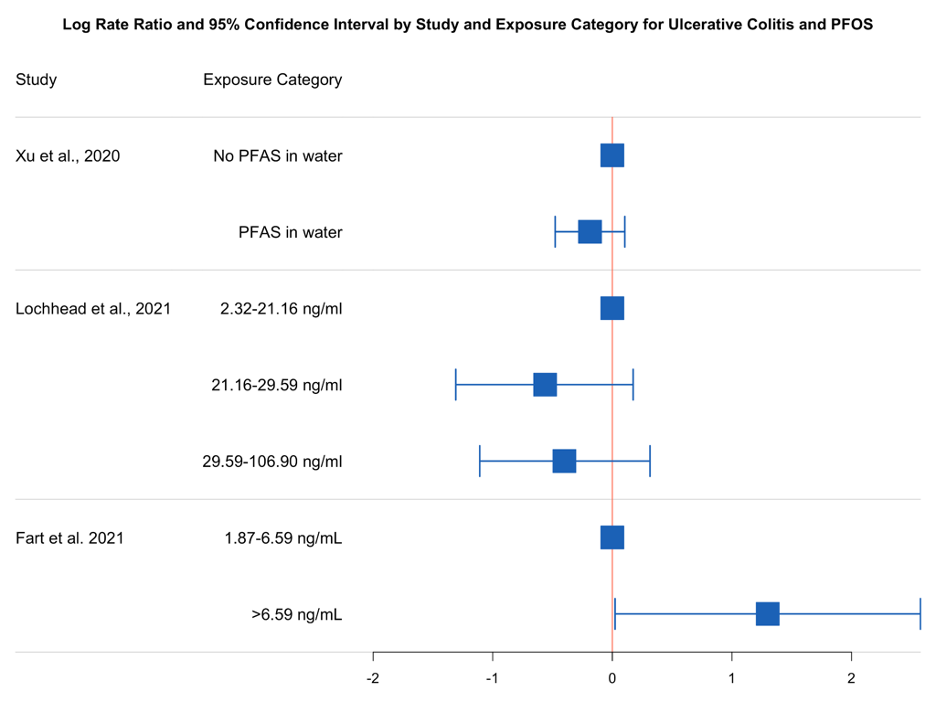


**Figure S6.** Log rate ratios for human studies of PFOS and Crohn’s disease


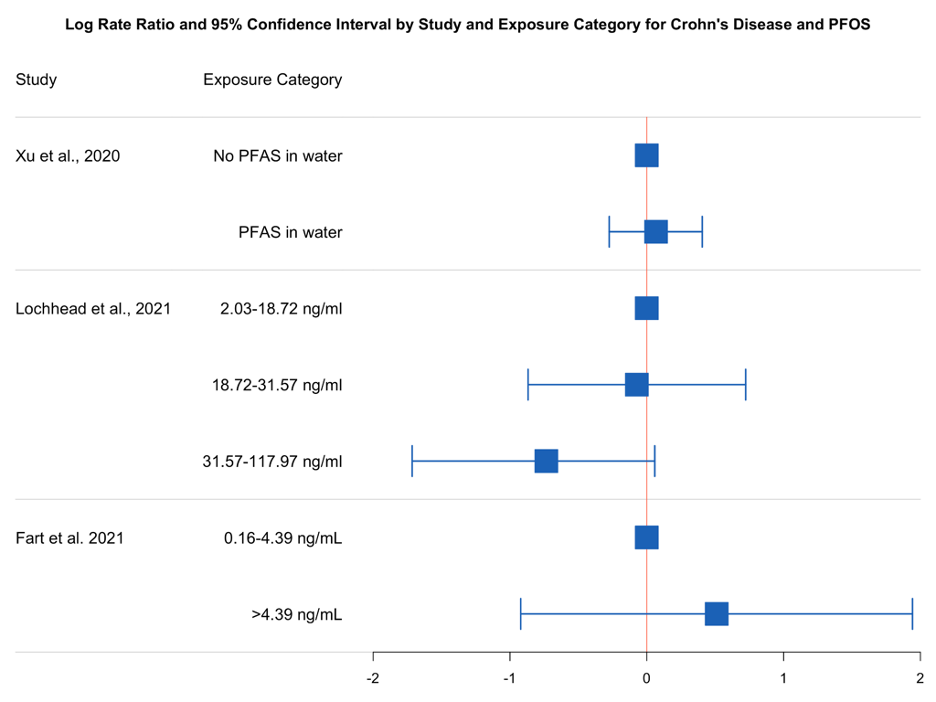


**Figure S7.** Log rate ratios for human studies of PFNA and ulcerative colitis


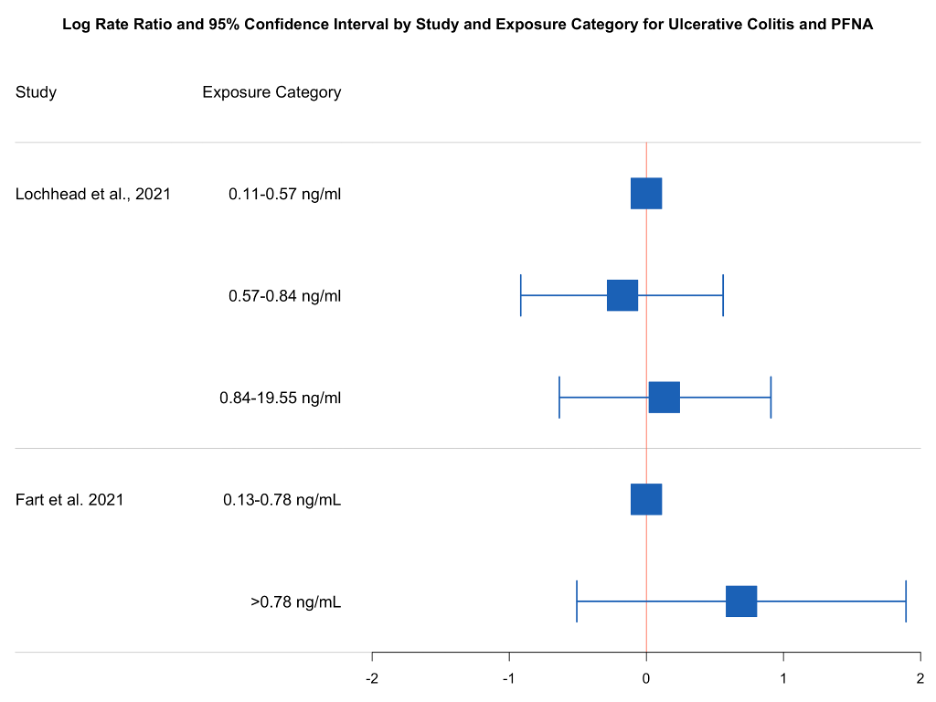


**Figure S8.**  Log rate ratios for human studies of PFNA and Crohn’s disease


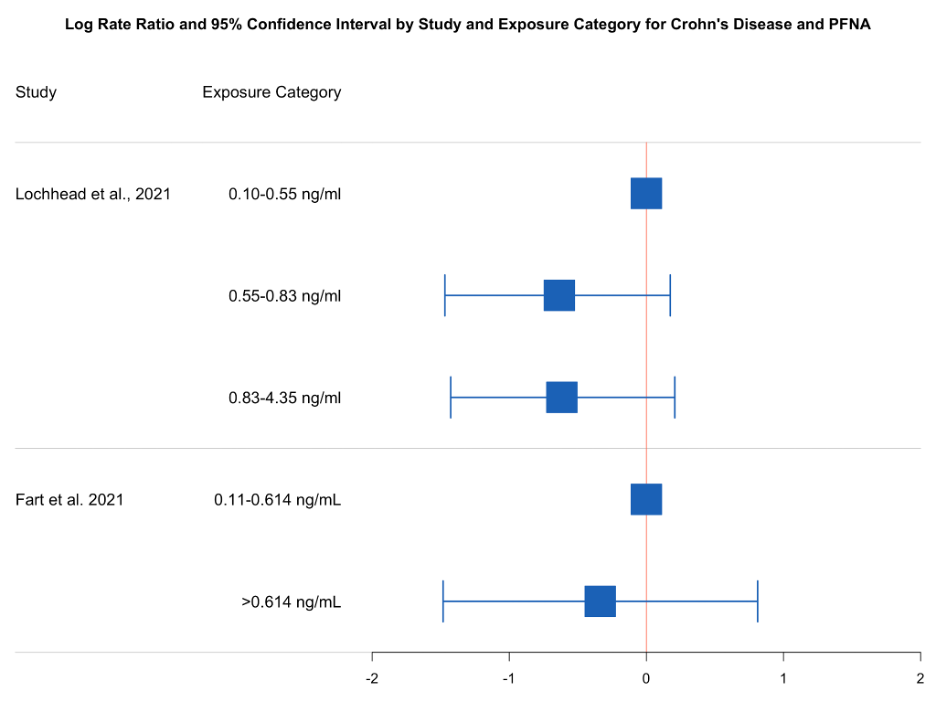


**Figure S9.** Log rate ratios for human studies of PFDA and ulcerative colitis


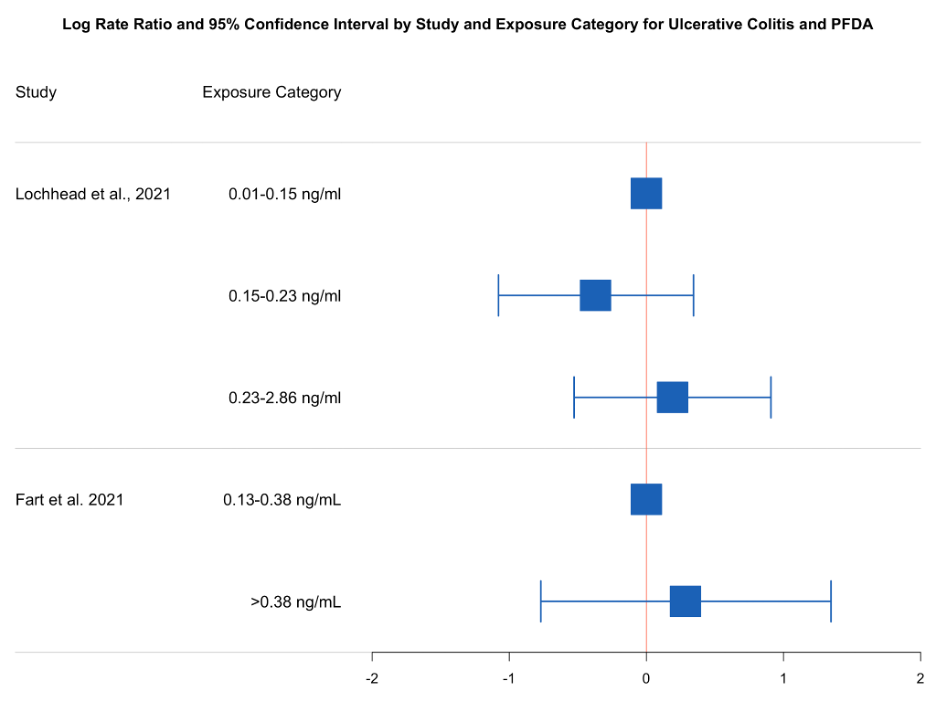


**Figure S10.** Log rate ratios for human studies of PFDA and Crohn’s disease


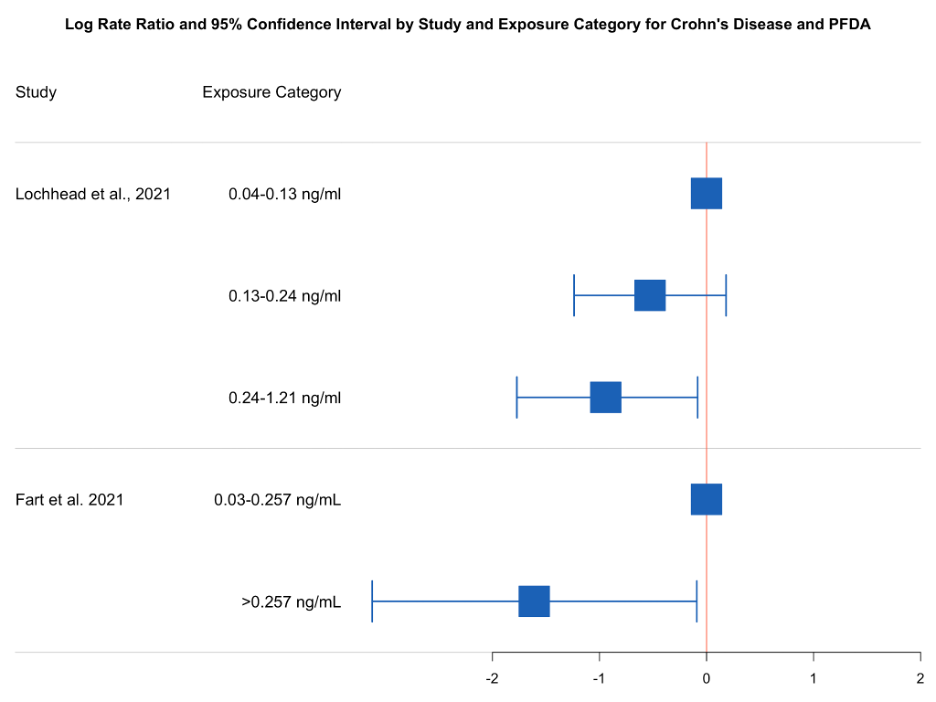


**IBD Manuscript R Code:**

#PFOA and Ulcerative Colitis

fartuc <- read.csv("/Users/celinaphillipson/Dropbox/Education/IBD Manuscript/R/Fart/FartUC.csv")

library(survival)

fartuc$cat_PFOA <- cut(fartuc$PFOA,

breaks=c(0, median(fartuc$PFOA), Inf),

labels = c('1', '2'))

fit.clr <- clogit(Case ~ cat_PFOA + strata(Pair.number), data = fartuc)

OR.CI <- cbind("OR" = exp(coef(fit.clr)), exp(confint(fit.clr)))

round(OR.CI, 3)

summary(fartuc$cat_PFOA)

summary(fit.clr) #results used in fart2021 dataframe

ucpfoa = list(

steenland2013 = data.frame(

stlab = c("Steenland et al., 2013","","",""),

labs = c("0-3.16 ng/mL-year","3.16-11.72 ng/mL-year", "11.72-70 ng/mL-year",">70 ng/mL-year"),

cutpoints = c(0, 3.16, 11.72, 70),

RR = c(1, 1.76, 2.63, 2.86),

lower = c(1, 1.04, 1.56, 1.65),

upper = c(1, 2.99, 4.43, 4.96)),

steenland2018 = data.frame(

stlab = c("Steenland et al., 2018","","","",""),

labs = c("0-1.01 ng/mL", "1.01-1.95 ng/mL","1.95-2.77 ng/mL", "2.77-4.72 ng/mL", ">4.72 ng/mL"),

cutpoints = c(0, 1.01, 1.95, 2.77, 4.72),

RR = c(1.0, 0.668, 36.23, 29.769, 2.834),

lower = c(1.0, 0.174, 9.705, 8.812, 0.913),

upper = c(1.0, 2.573, 135.274, 108.304, 8.801)),

lochhead2021 = data.frame(

stlab = c("Lochhead et al., 2021","",""),

labs = c("0.34-3.39 ng/mL","3.39-4.79 ng/mL","4.79-23.39 ng/mL"),

cutpoints = c(0.34, 3.39, 4.79),

RR = c(1.00, 1.01, 0.78),

lower = c(1.00, 0.51, 0.38),

upper = c(1.00, 2.02, 1.59)),

steenland2015 = data.frame(

stlab = c("Steenland et al., 2015","","",""),

labs = c("0-3030 ng/mL-year", "3030-6160 ng/mL-year", "6160-11420 ng/mL-year", "11420 ng/mL-year"),

cutpoints = c(0, 3030, 6160, 11420)/29.9, #avg age of diagnosis, average age of diagnosis in America

RR = c(1.0, 1.57, 0.57, 2.74),

lower = c(1.00, 0.52, 0.1, 0.78),

upper = c(1.00, 4.76, 3.11, 9.65)),

fart2021 = data.frame (

stlab = c("Fart el al., 2021", ""),

labs = c("0.61-1.96 ng/mL", ">1.96 ng/mL"),

cutpoints = c(0.61, 1.96),

RR = c(1.00, 1.8),

lower = c(1.00, 0.6032),

upper = c(1.00, 5.371)

)

)

#calculate midpoints

ucpfoa2 = lapply(ucpfoa, function(df) {

cp = df$cutpoints

l = length(cp) + 1

cp[l] = 4 * cp[l-1] # assume max is 4*last cutpoint

df$mids = apply(rbind(cp[-l],cp[-1]),2,mean)

return(df)

})

#subbing maximum in ucpfoa2

ucpfoa2$lochhead2021$mids[3] = mean(c(4.79,23.39))

ucpfoa2$fart2021$mids[2] = mean(c(1.96,14.84))

se = (log(4.96)-log(2.86))/qnorm(.975); 2*(1-pnorm(log(2.86),0,se)) # Steenland 2013

se = (log(8.801)-log(2.834))/qnorm(.975); 2*(1-pnorm(log(2.834),0,se)) # Steenland 2018

se = (log(1.59)-log(0.78))/qnorm(.975); 2*(1-pnorm(log(0.78),0,se)) # Lochhead 2021

se = (log(9.65)-log(2.74))/qnorm(.975); 2*(1-pnorm(log(2.74),0,se)) # Steenland 2015

se = (log(5.731)-log(1.80))/qnorm(.975); 2*(1-pnorm(log(1.80),0,se)) # Fart 2021

trendp = function(datalist){

4

lapply(datalist, function(df) {

se1 = (log(df$upper)-log(df$RR))/qnorm(.975) # se of log RR for each dose category

se2 = (log(df$RR)-log(df$lower))/qnorm(.975)

se = (se1 + se2) / 2

scores = 0:(length(se)-1)

if(se[1] == 0) {

lm1 = lm(log(RR) ~ 0 + scores, weights = 1/se^2, data=df, subset=se>0)

lm2 = lm(log(RR) ~ 0 + mids, weights = 1 / se^2, data=df, subset=se>0)

p1 = summary(lm1)$coef[1,4]

p2 = summary(lm2)$coef[1,4]

slope = summary(lm2)$coef[1,1]

se = summary(lm2)$coef[1,2]

} else {

lm1 = lm(log(RR) ~ scores, weights = 1 / se^2, data=df)

lm2 = lm(log(RR) ~ mids, weights = 1 / se^2, data=df)

p1 = summary(lm1)$coef[2,4]

p2 = summary(lm2)$coef[2,4]

slope = summary(lm2)$coef[2,1]

se = summary(lm2)$coef[2,2]

}

return(c(p1,p2,slope,se))

})

}

(ktrend = trendp(ucpfoa2))

fit.clr <- clogit(Case ~ PFOA + strata(Pair.number), data = fartuc)

ktrend$fart2021[3] <- coef(fit.clr)[1]

(coef(fit.clr))

ktrend$fart2021[4] <- (summary(fit.clr))$coef[3]

lapply(ktrend, function(df) round(exp(10*df[3] + 10*c(0,-1,1)*qnorm(.975)*df[4]),2))

library(meta)

klogRR = c(ktrend$steenland2013[3], ktrend$steenland2018[3], ktrend$lochhead2021[3], ktrend$steenland2015[3], ktrend$fart2021[3])

kse = c(ktrend$steenland2013[4], ktrend$steenland2018[4], ktrend$lochhead2021[4], ktrend$steenland2015[4], ktrend$fart2021[4])

(m2 = metagen(klogRR,kse))

round(exp(c(m2$TE.fixed,m2$lower.fixed,m2$upper.fixed)),3)

round(exp(c(m2$TE.random,m2$lower.random,m2$upper.random)),2)

### sensitivity analysis

(m2a = metagen(klogRR[c(4,5)], kse[c(4,5)]))

round(exp(c(m2a$TE.fixed,m2a$lower.fixed,m2a$upper.fixed)),2)

round(exp(c(m2a$TE.random,m2a$lower.random,m2a$upper.random)),2)

library(forestplot)

krows = unlist(lapply(ucpfoa2,function(df) dim(df)[1]))

kRR = unlist(lapply(ucpfoa2,function(df) df$RR))

klower = unlist(lapply(ucpfoa2,function(df) df$lower))

kupper = unlist(lapply(ucpfoa2,function(df) df$upper))

kstlabs = unlist(lapply(ucpfoa2,function(df) df$stlab))

klabs = unlist(lapply(ucpfoa2,function(df) df$labs))

klabels = cbind(c("Study", as.character(kstlabs)),

c("Exposure Category", as.character(klabs)))

png("ucpfoa.png", width = 12, height = 9, units = 'in', res = 300)

# ltg = "#99999922"

ltg = "gray80"

forestplot(labeltext=klabels, graph.pos=3,

mean=c(NA, log(kRR)),

lower=c(NA, log(klower)),

upper=c(NA, log(kupper)),

title="Log Rate Ratio and 95% Confidence Interval by Study and Exposure Category for Ulcerative Colitis and PFOA",

hrzl_lines=list("2" = gpar(lwd=1, col=ltg),"6" = gpar(lwd=1, col=ltg),

"6" = gpar(lwd=1, col=ltg), "11" = gpar(lwd=1, col=ltg),

"11" = gpar(lwd=1, col=ltg), "14" = gpar(lwd=1, col=ltg),

"18" = gpar(lwd=1, col=ltg)),

xticks=-3:5,

txt_gp=fpTxtGp(label=gpar(cex=1.25),

ticks=gpar(cex=1.1),

xlab=gpar(cex = 1.2),

title=gpar(cex = 1.2)),

col=fpColors(box="#1c61b6", lines="#1c61b6", zero = "tomato"),

zero=0, cex=0.9, lineheight = "auto", boxsize=0.3, colgap=unit(10,"mm"),

lwd.ci=2, ci.vertices=TRUE, ci.vertices.height = 0.2)

dev.off()

#PFOA and Crohn's Disease

fartcd <- read.csv("/Users/celinaphillipson/Dropbox/Education/IBD Manuscript/R/Fart/FartCD3.csv")

median(fartcd$PFOA)

summary(fartcd$PFOA)

fartcd$cat_PFOA2 <- cut(fartcd$PFOA,

breaks=c(0, median(fartcd$PFOA), Inf),

labels = c('1','2'))

fit.clr <- clogit(Case ~ cat_PFOA2 + strata(Pair.number), data = fartcd)

OR.CI <- cbind("OR" = exp(coef(fit.clr)), exp(confint(fit.clr)))

round(OR.CI, 3)

summary(fartcd$cat_PFOA2)

summary(fit.clr) #results used in fart2021 dataframe

cdpfoa = list(

steenland2013 = data.frame(

stlab = c("Steenland et al., 2013", "", "", ""),

labs = c("0-2.00 ng/mL-year","2.00-4.04 ng/mL-year", "4.04-18.36 ng/mL-year",">18.36 ng/mL-year"), #cutpoints provided via email: 99.8, 202, 918 (assuming cumulative so / 50 for average age of participants)

cutpoints = c(0, 2, 4.04, 18.36),

RR = c(1, 1.25, 1.15, 1.00),

lower = c(1, 0.61, 0.55, 0.48),

upper = c(1, 2.58, 2.41, 2.09)),

lochhead2021 = data.frame(

stlab = c("Lochhead et al., 2021","",""),

labs = c("0.89-3.36 ng/mL","3.36-4.84 ng/mL","4.84-73.12 ng/mL"),

cutpoints = c(0.89, 3.36, 4.84),

RR = c(1.00, 0.54, 0.59),

lower = c(1.00, 0.25, 0.28),

upper= c(1.00, 1.16, 1.25)),

fart2021 = data.frame(

stlab = c("Fart et al., 2021",""),

labs = c("0.47-1.72 ng/mL", ">1.72 ng/mL"),

cutpoints = c(0.47, 1.72),

RR = c(1.0, 2.33),

lower = c(1, 0.603),

upper = c(1, 9.023))

)

cdpfoa2 = lapply(cdpfoa, function(df) {

cp = df$cutpoints

l = length(cp) + 1

cp[l] = 4 * cp[l-1] # assume max is 4*last cutpoint

df$mids = apply(rbind(cp[-l],cp[-1]),2,mean)

return(df)

})

cdpfoa2$lochhead2021$mids[3] = mean(c(4.84,73.12))

cdpfoa2$fart2021$mids[2] = mean(c(1.72,7.30))

trendp = function(datalist){

3

lapply(datalist, function(df) {

se1 = (log(df$upper)-log(df$RR))/qnorm(.975) # se of log RR for each dose category

se2 = (log(df$RR)-log(df$lower))/qnorm(.975)

se = (se1 + se2) / 2

scores = 0:(length(se)-1)

if(se[1] == 0) {

lm1 = lm(log(RR) ~ 0 + scores, weights = 1/se^2, data=df, subset=se>0)

lm2 = lm(log(RR) ~ 0 + mids, weights = 1 / se^2, data=df, subset=se>0)

p1 = summary(lm1)$coef[1,4]

p2 = summary(lm2)$coef[1,4]

slope = summary(lm2)$coef[1,1]

se = summary(lm2)$coef[1,2]

} else {

lm1 = lm(log(RR) ~ scores, weights = 1 / se^2, data=df)

lm2 = lm(log(RR) ~ mids, weights = 1 / se^2, data=df)

p1 = summary(lm1)$coef[2,4]

p2 = summary(lm2)$coef[2,4]

slope = summary(lm2)$coef[2,1]

se = summary(lm2)$coef[2,2]

}

return(c(p1,p2,slope,se))

})

}

(ktrend = trendp(cdpfoa2))

fit.clr <- clogit(Case ~ PFOA + strata(Pair.number), data = fartcd)

ktrend$fart2021[3] <- coef(fit.clr)[1]

(coef(fit.clr))

ktrend$fart2021[4] <- (summary(fit.clr))$coef[3]

klogRR = c(ktrend$steenland2013[3], ktrend$lochhead2021[3], ktrend$fart2021[3])

kse = c(ktrend$steenland2013[4], ktrend$lochhead2021[4], ktrend$fart2021[4])

(m2 = metagen(klogRR,kse))

round(exp(c(m2$TE.fixed,m2$lower.fixed,m2$upper.fixed)),2)

round(exp(c(m2$TE.random,m2$lower.random,m2$upper.random)),2)

krows = unlist(lapply(cdpfoa2,function(df) dim(df)[1]))

kRR = unlist(lapply(cdpfoa2,function(df) df$RR))

klower = unlist(lapply(cdpfoa2,function(df) df$lower))

kupper = unlist(lapply(cdpfoa2,function(df) df$upper))

kstlabs = unlist(lapply(cdpfoa2,function(df) df$stlab))

klabs = unlist(lapply(cdpfoa2,function(df) df$labs))

klabels = cbind(c("Study", as.character(kstlabs)),

c("Exposure Category", as.character(klabs)))

png("cdpfoa.png", width = 12, height = 9, units = 'in', res = 300)

ltg = "gray80"

forestplot(labeltext=klabels, graph.pos=3,

mean=c(NA, log(kRR)),

lower=c(NA, log(klower)),

upper=c(NA, log(kupper)),

title="Log Rate Ratio and 95% Confidence Interval by Study and Exposure Category for Crohn's Disease and PFOA",

hrzl_lines=list("2" = gpar(lwd=1, col=ltg),"6" = gpar(lwd=1, col=ltg), "9" = gpar(lwd=1, col=ltg),

"11" = gpar(lwd=1, col=ltg)),

xticks=-2:2,

txt_gp=fpTxtGp(label=gpar(cex=1.25),

ticks=gpar(cex=1.1),

xlab=gpar(cex = 1.2),

title=gpar(cex = 1.2)),

col=fpColors(box="#1c61b6", lines="#1c61b6", zero = "tomato"),

zero=0, cex=0.9, lineheight = "auto", boxsize=0.3, colgap=unit(10,"mm"),

lwd.ci=2, ci.vertices=TRUE, ci.vertices.height = 0.2)

dev.off()

#PFHxS and Ulcerative Colitis

median(fartuc$PFHxS)

fartuc$cat_PFHxS <- cut(fartuc$PFHxS,

breaks=c(0, median(fartuc$PFHxS), Inf),

labels = c('1', '2'))

fit.clr <- clogit(Case ~ cat_PFHxS + strata(Pair.number), data = fartuc)

OR.CI <- cbind("OR" = exp(coef(fit.clr)), exp(confint(fit.clr)))

round(OR.CI, 3)

summary(fartuc$cat_PFHxS)

summary(fit.clr) #results used in fart2021 dataframe

ucpfhxs = list(

xu2020 = data.frame(

stlab = c("Xu et al., 2020",""),

labs = c("No PFAS in water", "PFAS in water"),

cutpoints = c(0, 1),

RR = c(1.0, 0.83),

lower = c(1.00, 0.62),

upper = c(1.00, 1.11)),

lochhead2021 = data.frame(

stlab = c("Lochhead et al., 2021","",""),

labs = c("0.20-1.47 ng/ml","1.47-2.69 ng/ml","2.69-47.48 ng/ml"),

cutpoints = c(0.20, 1.47, 2.69),

RR = c(1.00, 0.87, 0.62),

lower = c(1.00, 0.42, 0.29),

upper= c(1.00, 1.80, 1.31)),

fart2021 = data.frame(

stlab = c("Fart et al., 2021",""),

labs = c("0.17-0.78 ng/mL", ">0.78 ng/mL"),

cutpoints = c(0, 1),

RR = c(1, 1.40),

lower = c(1, 0.44),

upper = c(1, 4.41))

)

ucpfhxs2 = lapply(ucpfhxs, function(df) {

cp = df$cutpoints

l = length(cp) + 1

cp[l] = 4 * cp[l-1] # assume max is 4*last cutpoint

df$mids = apply(rbind(cp[-l],cp[-1]),2,mean)

return(df)

})

ucpfhxs2$lochhead2021$mids[3] = mean(c(2.69,47.48))

ucpfhxs2$fart2021$mids[2] = mean(c(0.78,5.80))

trendp = function(datalist){

3

lapply(datalist, function(df) {

se1 = (log(df$upper)-log(df$RR))/qnorm(.975) # se of log RR for each dose category

se2 = (log(df$RR)-log(df$lower))/qnorm(.975)

se = (se1 + se2) / 2

scores = 0:(length(se)-1)

if(se[1] == 0) {

lm1 = lm(log(RR) ~ 0 + scores, weights = 1/se^2, data=df, subset=se>0)

lm2 = lm(log(RR) ~ 0 + mids, weights = 1 / se^2, data=df, subset=se>0)

p1 = summary(lm1)$coef[1,4]

p2 = summary(lm2)$coef[1,4]

slope = summary(lm2)$coef[1,1]

se = summary(lm2)$coef[1,2]

} else {

lm1 = lm(log(RR) ~ scores, weights = 1 / se^2, data=df)

lm2 = lm(log(RR) ~ mids, weights = 1 / se^2, data=df)

p1 = summary(lm1)$coef[2,4]

p2 = summary(lm2)$coef[2,4]

slope = summary(lm2)$coef[2,1]

se = summary(lm2)$coef[2,2]

}

return(c(p1,p2,slope,se))

})

}

(ktrend = trendp(ucpfhxs2))

fit.clr <- clogit(Case ~ PFHxS + strata(Pair.number), data = fartuc)

ktrend$fart2021[3] <- coef(fit.clr)[1]

(coef(fit.clr))

ktrend$fart2021[4] <- (summary(fit.clr))$coef[3]

klogRR = c(ktrend$lochhead2021[3], ktrend$fart2021[3])

kse = c(ktrend$lochhead2021[4], ktrend$fart2021[4])

(m2 = metagen(klogRR,kse))

round(exp(c(m2$TE.fixed,m2$lower.fixed,m2$upper.fixed)),2)

round(exp(c(m2$TE.random,m2$lower.random,m2$upper.random)),2)

krows = unlist(lapply(ucpfhxs2,function(df) dim(df)[1]))

kRR = unlist(lapply(ucpfhxs2,function(df) df$RR))

klower = unlist(lapply(ucpfhxs2,function(df) df$lower))

kupper = unlist(lapply(ucpfhxs2,function(df) df$upper))

kstlabs = unlist(lapply(ucpfhxs2,function(df) df$stlab))

klabs = unlist(lapply(ucpfhxs2,function(df) df$labs))

klabels = cbind(c("Study", as.character(kstlabs)),

c("Exposure Category", as.character(klabs)))

png("ucpfhxs.png", width = 12, height = 9, units = 'in', res = 300)

ltg = "gray80"

forestplot(labeltext=klabels, graph.pos=3,

mean=c(NA, log(kRR)),

lower=c(NA, log(klower)),

upper=c(NA, log(kupper)),

title="Log Rate Ratio and 95% Confidence Interval by Study and Exposure Category for Ulcerative Colitis and PFHxS",

hrzl_lines=list("2" = gpar(lwd=1, col=ltg),"4" = gpar(lwd=1, col=ltg), "7" = gpar(lwd=1, col=ltg),"9" = gpar(lwd=1, col=ltg)),

xticks=-2:2,

txt_gp=fpTxtGp(label=gpar(cex=1.25),

ticks=gpar(cex=1.1),

xlab=gpar(cex = 1.2),

title=gpar(cex = 1.2)),

col=fpColors(box="#1c61b6", lines="#1c61b6", zero = "tomato"),

zero=0, cex=0.9, lineheight = "auto", boxsize=0.3, colgap=unit(10,"mm"),

lwd.ci=2, ci.vertices=TRUE, ci.vertices.height = 0.2)

dev.off()

#PFHxS and Crohn's Disease

median(fartcd$PFHxS)

fartcd$cat_PFHxS <- cut(fartcd$PFHxS,

breaks=c(0, median(fartcd$PFHxS), Inf),

labels = c('1', '2'))

fit.clr <- clogit(Case ~ cat_PFHxS + strata(Pair.number), data = fartcd)

OR.CI <- cbind("OR" = exp(coef(fit.clr)), exp(confint(fit.clr)))

round(OR.CI, 3)

summary(fartcd$cat_PFHxS)

summary(fit.clr) #results used in fart2021 dataframe

cdpfhxs = list(

xu2020 = data.frame(

stlab = c("Xu et al., 2020",""),

labs = c("No PFAS in water", "PFAS in water"),

cutpoints = c(0, 1),

RR = c(1.0, 1.07),

lower = c(1.00, 0.76),

upper = c(1.00, 1.50)),

lochhead2021 = data.frame(

stlab = c("Lochhead et al., 2021","",""),

labs = c("0.17-1.29 ng/ml","1.29-2.33 ng/ml","2.33-85.28 ng/ml"),

cutpoints = c(0.17, 1.29, 2.33),

RR = c(1.00, 0.77, 0.63),

lower = c(1.00, 0.36, 0.29),

upper= c(1.00, 1.63, 1.36)),

fart2021 = data.frame(

stlab = c("Fart et al., 2021",""),

labs = c("0.16-0.80 ng/mL", ">0.80 ng/mL"),

cutpoints = c(0, 1),

RR = c(1, 3.0),

lower = c(1, 0.606),

upper = c(1, 14.86))

)

cdpfhxs2 = lapply(cdpfhxs, function(df) {

cp = df$cutpoints

l = length(cp) + 1

cp[l] = 4 * cp[l-1] # assume max is 4*last cutpoint

df$mids = apply(rbind(cp[-l],cp[-1]),2,mean)

return(df)

})

cdpfhxs2$lochhead2021$mids[3] = mean(c(2.33,85.28))

cdpfhxs2$fart2021$mids[2] = mean(c(0.80,2.98))

trendp = function(datalist){

3

lapply(datalist, function(df) {

se1 = (log(df$upper)-log(df$RR))/qnorm(.975) # se of log RR for each dose category

se2 = (log(df$RR)-log(df$lower))/qnorm(.975)

se = (se1 + se2) / 2

scores = 0:(length(se)-1)

if(se[1] == 0) {

lm1 = lm(log(RR) ~ 0 + scores, weights = 1/se^2, data=df, subset=se>0)

lm2 = lm(log(RR) ~ 0 + mids, weights = 1 / se^2, data=df, subset=se>0)

p1 = summary(lm1)$coef[1,4]

p2 = summary(lm2)$coef[1,4]

slope = summary(lm2)$coef[1,1]

se = summary(lm2)$coef[1,2]

} else {

lm1 = lm(log(RR) ~ scores, weights = 1 / se^2, data=df)

lm2 = lm(log(RR) ~ mids, weights = 1 / se^2, data=df)

p1 = summary(lm1)$coef[2,4]

p2 = summary(lm2)$coef[2,4]

slope = summary(lm2)$coef[2,1]

se = summary(lm2)$coef[2,2]

}

return(c(p1,p2,slope,se))

})

}

(ktrend = trendp(cdpfhxs2))

fit.clr <- clogit(Case ~ PFHxS + strata(Pair.number), data = fartcd)

ktrend$fart2021[3] <- coef(fit.clr)[1]

(coef(fit.clr))

ktrend$fart2021[4] <- (summary(fit.clr))$coef[3]

klogRR = c(ktrend$lochhead2021[3], ktrend$fart2021[3])

kse = c(ktrend$lochhead2021[4], ktrend$fart2021[4])

(m2 = metagen(klogRR,kse))

round(exp(c(m2$TE.fixed,m2$lower.fixed,m2$upper.fixed)),2)

round(exp(c(m2$TE.random,m2$lower.random,m2$upper.random)),2)

krows = unlist(lapply(cdpfhxs2,function(df) dim(df)[1]))

kRR = unlist(lapply(cdpfhxs2,function(df) df$RR))

klower = unlist(lapply(cdpfhxs2,function(df) df$lower))

kupper = unlist(lapply(cdpfhxs2,function(df) df$upper))

kstlabs = unlist(lapply(cdpfhxs2,function(df) df$stlab))

klabs = unlist(lapply(cdpfhxs2,function(df) df$labs))

klabels = cbind(c("Study", as.character(kstlabs)),

c("Exposure Category", as.character(klabs)))

png("cdpfhxs.png", width = 12, height = 9, units = 'in', res = 300)

ltg = "gray80"

forestplot(labeltext=klabels, graph.pos=3,

mean=c(NA, log(kRR)),

lower=c(NA, log(klower)),

upper=c(NA, log(kupper)),

title="Log Rate Ratio and 95% Confidence Interval by Study and Exposure Category for Crohn's Disease and PFHxS",

hrzl_lines=list("2" = gpar(lwd=1, col=ltg),"4" = gpar(lwd=1, col=ltg), "7" = gpar(lwd=1, col=ltg),"9" = gpar(lwd=1, col=ltg)),

xticks=-2:2,

txt_gp=fpTxtGp(label=gpar(cex=1.25),

ticks=gpar(cex=1.1),

xlab=gpar(cex = 1.2),

title=gpar(cex = 1.2)),

col=fpColors(box="#1c61b6", lines="#1c61b6", zero = "tomato"),

zero=0, cex=0.9, lineheight = "auto", boxsize=0.3, colgap=unit(10,"mm"),

lwd.ci=2, ci.vertices=TRUE, ci.vertices.height = 0.2)

dev.off()

#PFOS and Ulcerative Colitis

median(fartuc$L.PFOS)

fartuc$cat_L.PFOS <- cut(fartuc$L.PFOS,

breaks=c(0, median(fartuc$L.PFOS), Inf),

labels = c('1', '2'))

fit.clr <- clogit(Case ~ cat_L.PFOS + strata(Pair.number), data = fartuc)

OR.CI <- cbind("OR" = exp(coef(fit.clr)), exp(confint(fit.clr)))

round(OR.CI, 3)

summary(fartuc$cat_L.PFOS)

summary(fit.clr) #results used in fart2021 dataframe

ucpfos = list(

xu2020 = data.frame(

stlab = c("Xu et al., 2020",""),

labs = c("No PFAS in water", "PFAS in water"),

cutpoints = c(0, 1),

RR = c(1.0, 0.83),

lower = c(1.00, 0.62),

upper = c(1.00, 1.11)),

lochhead2021 = data.frame(

stlab = c("Lochhead et al., 2021","",""),

labs = c("2.32-21.16 ng/ml","21.16-29.59 ng/ml","29.59-106.90 ng/ml"),

cutpoints = c(2.32, 21.16, 29.59),

RR = c(1.00, 0.57, 0.67),

lower = c(1.00, 0.27, 0.33),

upper= c(1.00, 1.19, 1.37)),

fart2021 = data.frame(

stlab = c("Fart et al., 2021",""),

labs = c("1.87-6.59 ng/mL", ">6.59 ng/mL"),

cutpoints = c(0, 1),

RR = c(1, 3.667),

lower = c(1, 1.023),

upper = c(1, 13.143))

)

ucpfos2 = lapply(ucpfos, function(df) {

cp = df$cutpoints

l = length(cp) + 1

cp[l] = 4 * cp[l-1] # assume max is 4*last cutpoint

df$mids = apply(rbind(cp[-l],cp[-1]),2,mean)

return(df)

})

ucpfos2$lochhead2021$mids[3] = mean(c(29.59,106.90))

ucpfos2$fart2021$mids[2] = mean(c(6.59,111.87))

trendp = function(datalist){

3

lapply(datalist, function(df) {

se1 = (log(df$upper)-log(df$RR))/qnorm(.975) # se of log RR for each dose category

se2 = (log(df$RR)-log(df$lower))/qnorm(.975)

se = (se1 + se2) / 2

scores = 0:(length(se)-1)

if(se[1] == 0) {

lm1 = lm(log(RR) ~ 0 + scores, weights = 1/se^2, data=df, subset=se>0)

lm2 = lm(log(RR) ~ 0 + mids, weights = 1 / se^2, data=df, subset=se>0)

p1 = summary(lm1)$coef[1,4]

p2 = summary(lm2)$coef[1,4]

slope = summary(lm2)$coef[1,1]

se = summary(lm2)$coef[1,2]

} else {

lm1 = lm(log(RR) ~ scores, weights = 1 / se^2, data=df)

lm2 = lm(log(RR) ~ mids, weights = 1 / se^2, data=df)

p1 = summary(lm1)$coef[2,4]

p2 = summary(lm2)$coef[2,4]

slope = summary(lm2)$coef[2,1]

se = summary(lm2)$coef[2,2]

}

return(c(p1,p2,slope,se))

})

}

(ktrend = trendp(ucpfos2))

fit.clr <- clogit(Case ~ L.PFOS + strata(Pair.number), data = fartuc)

ktrend$fart2021[3] <- coef(fit.clr)[1]

(coef(fit.clr))

ktrend$fart2021[4] <- (summary(fit.clr))$coef[3]

klogRR = c(ktrend$lochhead2021[3], ktrend$fart2021[3])

kse = c(ktrend$lochhead2021[4], ktrend$fart2021[4])

(m2 = metagen(klogRR,kse))

round(exp(c(m2$TE.fixed,m2$lower.fixed,m2$upper.fixed)),2)

round(exp(c(m2$TE.random,m2$lower.random,m2$upper.random)),2)

krows = unlist(lapply(ucpfos2,function(df) dim(df)[1]))

kRR = unlist(lapply(ucpfos2,function(df) df$RR))

klower = unlist(lapply(ucpfos2,function(df) df$lower))

kupper = unlist(lapply(ucpfos2,function(df) df$upper))

kstlabs = unlist(lapply(ucpfos2,function(df) df$stlab))

klabs = unlist(lapply(ucpfos2,function(df) df$labs))

klabels = cbind(c("Study", as.character(kstlabs)),

c("Exposure Category", as.character(klabs)))

png("ucpfos.png", width = 12, height = 9, units = 'in', res = 300)

ltg = "gray80"

forestplot(labeltext=klabels, graph.pos=3,

mean=c(NA, log(kRR)),

lower=c(NA, log(klower)),

upper=c(NA, log(kupper)),

title="Log Rate Ratio and 95% Confidence Interval by Study and Exposure Category for Ulcerative Colitis and PFOS",

hrzl_lines=list("2" = gpar(lwd=1, col=ltg),"4" = gpar(lwd=1, col=ltg), "7" = gpar(lwd=1, col=ltg),"9" = gpar(lwd=1, col=ltg)),

xticks=-2:2,

txt_gp=fpTxtGp(label=gpar(cex=1.25),

ticks=gpar(cex=1.1),

xlab=gpar(cex = 1.2),

title=gpar(cex = 1.2)),

col=fpColors(box="#1c61b6", lines="#1c61b6", zero = "tomato"),

zero=0, cex=0.9, lineheight = "auto", boxsize=0.3, colgap=unit(10,"mm"),

lwd.ci=2, ci.vertices=TRUE, ci.vertices.height = 0.2)

dev.off()

#PFOS and Crohn's Disease

median(fartcd$L.PFOS)

fartcd$cat_L.PFOS <- cut(fartcd$L.PFOS,

breaks=c(0, median(fartcd$L.PFOS), Inf),

labels = c('1', '2'))

fit.clr <- clogit(Case ~ cat_L.PFOS + strata(Pair.number), data = fartcd)

OR.CI <- cbind("OR" = exp(coef(fit.clr)), exp(confint(fit.clr)))

round(OR.CI, 3)

summary(fartcd$cat_L.PFOS)

summary(fit.clr) #results used in fart2021 dataframe

cdpfos = list(

xu2020 = data.frame(

stlab = c("Xu et al., 2020",""),

labs = c("No PFAS in water", "PFAS in water"),

cutpoints = c(0, 1),

RR = c(1.0, 1.07),

lower = c(1.00, 0.76),

upper = c(1.00, 1.50)),

lochhead2021 = data.frame(

stlab = c("Lochhead et al., 2021","",""),

labs = c("2.03-18.72 ng/ml","18.72-31.57 ng/ml","31.57-117.97 ng/ml"),

cutpoints = c(2.03, 18.72, 31.57),

RR = c(1.00, 0.93, 0.48),

lower = c(1.00, 0.42, 0.18),

upper= c(1.00, 2.06, 1.06)),

fart2021 = data.frame(

stlab = c("Fart et al., 2021",""),

labs = c("0.16-4.39 ng/mL", ">4.39 ng/mL"),

cutpoints = c(0, 1),

RR = c(1, 1.667),

lower = c(1, 0.398),

upper = c(1, 6.974))

)

cdpfos2 = lapply(cdpfos, function(df) {

cp = df$cutpoints

l = length(cp) + 1

cp[l] = 4 * cp[l-1] # assume max is 4*last cutpoint

df$mids = apply(rbind(cp[-l],cp[-1]),2,mean)

return(df)

})

cdpfos2$lochhead2021$mids[3] = mean(c(31.57,117.97))

cdpfos2$fart2021$mids[2] = mean(c(4.39,2.98))

trendp = function(datalist){

3

lapply(datalist, function(df) {

se1 = (log(df$upper)-log(df$RR))/qnorm(.975) # se of log RR for each dose category

se2 = (log(df$RR)-log(df$lower))/qnorm(.975)

se = (se1 + se2) / 2

scores = 0:(length(se)-1)

if(se[1] == 0) {

lm1 = lm(log(RR) ~ 0 + scores, weights = 1/se^2, data=df, subset=se>0)

lm2 = lm(log(RR) ~ 0 + mids, weights = 1 / se^2, data=df, subset=se>0)

p1 = summary(lm1)$coef[1,4]

p2 = summary(lm2)$coef[1,4]

slope = summary(lm2)$coef[1,1]

se = summary(lm2)$coef[1,2]

} else {

lm1 = lm(log(RR) ~ scores, weights = 1 / se^2, data=df)

lm2 = lm(log(RR) ~ mids, weights = 1 / se^2, data=df)

p1 = summary(lm1)$coef[2,4]

p2 = summary(lm2)$coef[2,4]

slope = summary(lm2)$coef[2,1]

se = summary(lm2)$coef[2,2]

}

return(c(p1,p2,slope,se))

})

}

(ktrend = trendp(cdpfos2))

fit.clr <- clogit(Case ~ L.PFOS + strata(Pair.number), data = fartcd)

ktrend$fart2021[3] <- coef(fit.clr)[1]

(coef(fit.clr))

ktrend$fart2021[4] <- (summary(fit.clr))$coef[3]

klogRR = c(ktrend$lochhead2021[3], ktrend$fart2021[3])

kse = c(ktrend$lochhead2021[4], ktrend$fart2021[4])

(m2 = metagen(klogRR,kse))

round(exp(c(m2$TE.fixed,m2$lower.fixed,m2$upper.fixed)),3)

round(exp(c(m2$TE.random,m2$lower.random,m2$upper.random)),2)

krows = unlist(lapply(cdpfos2,function(df) dim(df)[1]))

kRR = unlist(lapply(cdpfos2,function(df) df$RR))

klower = unlist(lapply(cdpfos2,function(df) df$lower))

kupper = unlist(lapply(cdpfos2,function(df) df$upper))

kstlabs = unlist(lapply(cdpfos2,function(df) df$stlab))

klabs = unlist(lapply(cdpfos2,function(df) df$labs))

klabels = cbind(c("Study", as.character(kstlabs)),

c("Exposure Category", as.character(klabs)))

png("cdpfos.png", width = 12, height = 9, units = 'in', res = 300)

ltg = "gray80"

forestplot(labeltext=klabels, graph.pos=3,

mean=c(NA, log(kRR)),

lower=c(NA, log(klower)),

upper=c(NA, log(kupper)),

title="Log Rate Ratio and 95% Confidence Interval by Study and Exposure Category for Crohn's Disease and PFOS",

hrzl_lines=list("2" = gpar(lwd=1, col=ltg),"4" = gpar(lwd=1, col=ltg), "7" = gpar(lwd=1, col=ltg),"9" = gpar(lwd=1, col=ltg)),

xticks=-2:2,

txt_gp=fpTxtGp(label=gpar(cex=1.25),

ticks=gpar(cex=1.1),

xlab=gpar(cex = 1.2),

title=gpar(cex = 1.2)),

col=fpColors(box="#1c61b6", lines="#1c61b6", zero = "tomato"),

zero=0, cex=0.9, lineheight = "auto", boxsize=0.3, colgap=unit(10,"mm"),

lwd.ci=2, ci.vertices=TRUE, ci.vertices.height = 0.2)

dev.off()

#PFNA and Ulcerative Colitis

median(fartuc$PFNA)

fartuc$cat_PFNA <- cut(fartuc$PFNA,

breaks=c(0, median(fartuc$PFNA), Inf),

labels = c('1', '2'))

fit.clr <- clogit(Case ~ cat_PFNA + strata(Pair.number), data = fartuc)

OR.CI <- cbind("OR" = exp(coef(fit.clr)), exp(confint(fit.clr)))

round(OR.CI, 3)

summary(fartuc$cat_PFNA)

summary(fit.clr) #results used in fart2021 dataframe

ucpfna = list(

lochhead2021 = data.frame(

stlab = c("Lochhead et al., 2021","",""),

labs = c("0.11-0.57 ng/ml","0.57-0.84 ng/ml","0.84-19.55 ng/ml"),

cutpoints = c(0.11, 0.69, 1.26),

RR = c(1.00, 0.84, 1.14),

lower = c(1.00, 0.40, 0.53),

upper= c(1.00, 1.75, 2.48)),

fart2021 = data.frame(

stlab = c("Fart et al., 2021",""),

labs = c("0.13-0.78 ng/mL", ">0.78 ng/mL"),

cutpoints = c(0, 1),

RR = c(1, 2.00),

lower = c(1, 0.602),

upper = c(1, 6.642))

)

ucpfna2 = lapply(ucpfna, function(df) {

cp = df$cutpoints

l = length(cp) + 1

cp[l] = 4 * cp[l-1] # assume max is 4*last cutpoint

df$mids = apply(rbind(cp[-l],cp[-1]),2,mean)

return(df)

})

ucpfna2$lochhead2021$mids[3] = mean(c(0.84,19.55))

ucpfna2$fart2021$mids[2] = mean(c(0.78,3.21))

trendp = function(datalist){

3

lapply(datalist, function(df) {

se1 = (log(df$upper)-log(df$RR))/qnorm(.975) # se of log RR for each dose category

se2 = (log(df$RR)-log(df$lower))/qnorm(.975)

se = (se1 + se2) / 2

scores = 0:(length(se)-1)

if(se[1] == 0) {

lm1 = lm(log(RR) ~ 0 + scores, weights = 1/se^2, data=df, subset=se>0)

lm2 = lm(log(RR) ~ 0 + mids, weights = 1 / se^2, data=df, subset=se>0)

p1 = summary(lm1)$coef[1,4]

p2 = summary(lm2)$coef[1,4]

slope = summary(lm2)$coef[1,1]

se = summary(lm2)$coef[1,2]

} else {

lm1 = lm(log(RR) ~ scores, weights = 1 / se^2, data=df)

lm2 = lm(log(RR) ~ mids, weights = 1 / se^2, data=df)

p1 = summary(lm1)$coef[2,4]

p2 = summary(lm2)$coef[2,4]

slope = summary(lm2)$coef[2,1]

se = summary(lm2)$coef[2,2]

}

return(c(p1,p2,slope,se))

})

}

(ktrend = trendp(ucpfna2))

fit.clr <- clogit(Case ~ PFNA + strata(Pair.number), data = fartuc)

ktrend$fart2021[3] <- coef(fit.clr)[1]

(coef(fit.clr))

ktrend$fart2021[4] <- (summary(fit.clr))$coef[3]

klogRR = c(ktrend$lochhead2021[3], ktrend$fart2021[3])

kse = c(ktrend$lochhead2021[4], ktrend$fart2021[4])

(m2 = metagen(klogRR,kse))

round(exp(c(m2$TE.fixed,m2$lower.fixed,m2$upper.fixed)),2)

round(exp(c(m2$TE.random,m2$lower.random,m2$upper.random)),2)

krows = unlist(lapply(ucpfna2,function(df) dim(df)[1]))

kRR = unlist(lapply(ucpfna2,function(df) df$RR))

klower = unlist(lapply(ucpfna2,function(df) df$lower))

kupper = unlist(lapply(ucpfna2,function(df) df$upper))

kstlabs = unlist(lapply(ucpfna2,function(df) df$stlab))

klabs = unlist(lapply(ucpfna2,function(df) df$labs))

klabels = cbind(c("Study", as.character(kstlabs)),

c("Exposure Category", as.character(klabs)))

png("ucpfna.png", width = 12, height = 9, units = 'in', res = 300)

ltg = "gray80"

forestplot(labeltext=klabels, graph.pos=3,

mean=c(NA, log(kRR)),

lower=c(NA, log(klower)),

upper=c(NA, log(kupper)),

title="Log Rate Ratio and 95% Confidence Interval by Study and Exposure Category for Ulcerative Colitis and PFNA",

hrzl_lines=list("2" = gpar(lwd=1, col=ltg),"5" = gpar(lwd=1, col=ltg), "7" = gpar(lwd=1, col=ltg)),

xticks=-2:2,

txt_gp=fpTxtGp(label=gpar(cex=1.25),

ticks=gpar(cex=1.1),

xlab=gpar(cex = 1.2),

title=gpar(cex = 1.2)),

col=fpColors(box="#1c61b6", lines="#1c61b6", zero = "tomato"),

zero=0, cex=0.9, lineheight = "auto", boxsize=0.3, colgap=unit(10,"mm"),

lwd.ci=2, ci.vertices=TRUE, ci.vertices.height = 0.2)

dev.off()

#PFNA and Crohn's Disease

median(fartcd$PFNA)

fartcd$cat_PFNA <- cut(fartcd$PFNA,

breaks=c(0, median(fartcd$PFNA), Inf),

labels = c('1', '2'))

fit.clr <- clogit(Case ~ cat_PFNA + strata(Pair.number), data = fartcd)

OR.CI <- cbind("OR" = exp(coef(fit.clr)), exp(confint(fit.clr)))

round(OR.CI, 3)

summary(fartcd$cat_PFNA)

summary(fit.clr) #results used in fart2021 dataframe

cdpfna = list(

lochhead2021 = data.frame(

stlab = c("Lochhead et al., 2021","",""),

labs = c("0.10-0.55 ng/ml","0.55-0.83 ng/ml","0.83-4.35 ng/ml"),

cutpoints = c(0.10, 0.55, 0.83),

RR = c(1.00, 0.53, 0.54),

lower = c(1.00, 0.23, 0.24),

upper= c(1.00, 1.19, 1.23)),

fart2021 = data.frame(

stlab = c("Fart et al., 2021",""),

labs = c("0.11-0.614 ng/mL", ">0.614 ng/mL"),

cutpoints = c(0, 1),

RR = c(1, 0.714),

lower = c(1, 0.227),

upper = c(1, 2.251))

)

cdpfna2 = lapply(cdpfna, function(df) {

cp = df$cutpoints

l = length(cp) + 1

cp[l] = 4 * cp[l-1] # assume max is 4*last cutpoint

df$mids = apply(rbind(cp[-l],cp[-1]),2,mean)

return(df)

})

cdpfna2$lochhead2021$mids[3] = mean(c(0.83,4.35))

cdpfna2$fart2021$mids[2] = mean(c(0.614,1.71))

trendp = function(datalist){

3

lapply(datalist, function(df) {

se1 = (log(df$upper)-log(df$RR))/qnorm(.975) # se of log RR for each dose category

se2 = (log(df$RR)-log(df$lower))/qnorm(.975)

se = (se1 + se2) / 2

scores = 0:(length(se)-1)

if(se[1] == 0) {

lm1 = lm(log(RR) ~ 0 + scores, weights = 1/se^2, data=df, subset=se>0)

lm2 = lm(log(RR) ~ 0 + mids, weights = 1 / se^2, data=df, subset=se>0)

p1 = summary(lm1)$coef[1,4]

p2 = summary(lm2)$coef[1,4]

slope = summary(lm2)$coef[1,1]

se = summary(lm2)$coef[1,2]

} else {

lm1 = lm(log(RR) ~ scores, weights = 1 / se^2, data=df)

lm2 = lm(log(RR) ~ mids, weights = 1 / se^2, data=df)

p1 = summary(lm1)$coef[2,4]

p2 = summary(lm2)$coef[2,4]

slope = summary(lm2)$coef[2,1]

se = summary(lm2)$coef[2,2]

}

return(c(p1,p2,slope,se))

})

}

(ktrend = trendp(cdpfna2))

fit.clr <- clogit(Case ~ PFNA + strata(Pair.number), data = fartcd)

ktrend$fart2021[3] <- coef(fit.clr)[1]

(coef(fit.clr))

ktrend$fart2021[4] <- (summary(fit.clr))$coef[3]

klogRR = c(ktrend$lochhead2021[3], ktrend$fart2021[3])

kse = c(ktrend$lochhead2021[4], ktrend$fart2021[4])

(m2 = metagen(klogRR,kse))

round(exp(c(m2$TE.fixed,m2$lower.fixed,m2$upper.fixed)),2)

round(exp(c(m2$TE.random,m2$lower.random,m2$upper.random)),2)

krows = unlist(lapply(cdpfna2,function(df) dim(df)[1]))

kRR = unlist(lapply(cdpfna2,function(df) df$RR))

klower = unlist(lapply(cdpfna2,function(df) df$lower))

kupper = unlist(lapply(cdpfna2,function(df) df$upper))

kstlabs = unlist(lapply(cdpfna2,function(df) df$stlab))

klabs = unlist(lapply(cdpfna2,function(df) df$labs))

klabels = cbind(c("Study", as.character(kstlabs)),

c("Exposure Category", as.character(klabs)))

png("cdpfna.png", width = 12, height = 9, units = 'in', res = 300)

ltg = "gray80"

forestplot(labeltext=klabels, graph.pos=3,

mean=c(NA, log(kRR)),

lower=c(NA, log(klower)),

upper=c(NA, log(kupper)),

title="Log Rate Ratio and 95% Confidence Interval by Study and Exposure Category for Crohn's Disease and PFNA",

hrzl_lines=list("2" = gpar(lwd=1, col=ltg),"5" = gpar(lwd=1, col=ltg), "7" = gpar(lwd=1, col=ltg)),

xticks=-2:2,

txt_gp=fpTxtGp(label=gpar(cex=1.25),

ticks=gpar(cex=1.1),

xlab=gpar(cex = 1.2),

title=gpar(cex = 1.2)),

col=fpColors(box="#1c61b6", lines="#1c61b6", zero = "tomato"),

zero=0, cex=0.9, lineheight = "auto", boxsize=0.3, colgap=unit(10,"mm"),

lwd.ci=2, ci.vertices=TRUE, ci.vertices.height = 0.2)

dev.off()

#PFDA and Ulcerative Colitis

median(fartuc$PFDA)

fartuc$cat_PFDA <- cut(fartuc$PFDA,

breaks=c(0, median(fartuc$PFDA), Inf),

labels = c('1', '2'))

fit.clr <- clogit(Case ~ cat_PFDA + strata(Pair.number), data = fartuc)

OR.CI <- cbind("OR" = exp(coef(fit.clr)), exp(confint(fit.clr)))

round(OR.CI, 3)

summary(fartuc$cat_PFDA)

summary(fit.clr) #results used in fart2021 dataframe

ucpfda = list(

lochhead2021 = data.frame(

stlab = c("Lochhead et al., 2021","",""),

labs = c("0.01-0.15 ng/ml","0.15-0.23 ng/ml","0.23-2.86 ng/ml"),

cutpoints = c(0.01, 0.15, 0.23),

RR = c(1.00, 0.69, 1.21),

lower = c(1.00, 0.34, 0.59),

upper= c(1.00, 1.41, 2.48)),

fart2021 = data.frame(

stlab = c("Fart et al., 2021",""),

labs = c("0.13-0.38 ng/mL", ">0.38 ng/mL"),

cutpoints = c(0, 1),

RR = c(1, 1.33),

lower = c(1, 0.463),

upper = c(1, 3.843))

)

ucpfda2 = lapply(ucpfda, function(df) {

cp = df$cutpoints

l = length(cp) + 1

cp[l] = 4 * cp[l-1] # assume max is 4*last cutpoint

df$mids = apply(rbind(cp[-l],cp[-1]),2,mean)

return(df)

})

ucpfda2$lochhead2021$mids[3] = mean(c(0.23,2.86))

ucpfda2$fart2021$mids[2] = mean(c(0.38,1.20))

trendp = function(datalist){

3

lapply(datalist, function(df) {

se1 = (log(df$upper)-log(df$RR))/qnorm(.975) # se of log RR for each dose category

se2 = (log(df$RR)-log(df$lower))/qnorm(.975)

se = (se1 + se2) / 2

scores = 0:(length(se)-1)

if(se[1] == 0) {

lm1 = lm(log(RR) ~ 0 + scores, weights = 1/se^2, data=df, subset=se>0)

lm2 = lm(log(RR) ~ 0 + mids, weights = 1 / se^2, data=df, subset=se>0)

p1 = summary(lm1)$coef[1,4]

p2 = summary(lm2)$coef[1,4]

slope = summary(lm2)$coef[1,1]

se = summary(lm2)$coef[1,2]

} else {

lm1 = lm(log(RR) ~ scores, weights = 1 / se^2, data=df)

lm2 = lm(log(RR) ~ mids, weights = 1 / se^2, data=df)

p1 = summary(lm1)$coef[2,4]

p2 = summary(lm2)$coef[2,4]

slope = summary(lm2)$coef[2,1]

se = summary(lm2)$coef[2,2]

}

return(c(p1,p2,slope,se))

})

}

(ktrend = trendp(ucpfda2))

fit.clr <- clogit(Case ~ PFDA + strata(Pair.number), data = fartuc)

ktrend$fart2021[3] <- coef(fit.clr)[1]

(coef(fit.clr))

ktrend$fart2021[4] <- (summary(fit.clr))$coef[3]

klogRR = c(ktrend$lochhead2021[3], ktrend$fart2021[3])

kse = c(ktrend$lochhead2021[4], ktrend$fart2021[4])

(m2 = metagen(klogRR,kse))

round(exp(c(m2$TE.fixed,m2$lower.fixed,m2$upper.fixed)),2)

round(exp(c(m2$TE.random,m2$lower.random,m2$upper.random)),2)

krows = unlist(lapply(ucpfda2,function(df) dim(df)[1]))

kRR = unlist(lapply(ucpfda2,function(df) df$RR))

klower = unlist(lapply(ucpfda2,function(df) df$lower))

kupper = unlist(lapply(ucpfda2,function(df) df$upper))

kstlabs = unlist(lapply(ucpfda2,function(df) df$stlab))

klabs = unlist(lapply(ucpfda2,function(df) df$labs))

klabels = cbind(c("Study", as.character(kstlabs)),

c("Exposure Category", as.character(klabs)))

png("ucpfda.png", width = 12, height = 9, units = 'in', res = 300)

ltg = "gray80"

forestplot(labeltext=klabels, graph.pos=3,

mean=c(NA, log(kRR)),

lower=c(NA, log(klower)),

upper=c(NA, log(kupper)),

title="Log Rate Ratio and 95% Confidence Interval by Study and Exposure Category for Ulcerative Colitis and PFDA",

hrzl_lines=list("2" = gpar(lwd=1, col=ltg),"5" = gpar(lwd=1, col=ltg), "7" = gpar(lwd=1, col=ltg)),

xticks=-2:2,

txt_gp=fpTxtGp(label=gpar(cex=1.25),

ticks=gpar(cex=1.1),

xlab=gpar(cex = 1.2),

title=gpar(cex = 1.2)),

col=fpColors(box="#1c61b6", lines="#1c61b6", zero = "tomato"),

zero=0, cex=0.9, lineheight = "auto", boxsize=0.3, colgap=unit(10,"mm"),

lwd.ci=2, ci.vertices=TRUE, ci.vertices.height = 0.2)

dev.off()

#PFDA and Crohn's Disease

median(fartcd$PFDA)

fartcd$cat_PFDA <- cut(fartcd$PFDA,

breaks=c(0, median(fartcd$PFDA), Inf),

labels = c('1', '2'))

fit.clr <- clogit(Case ~ cat_PFDA + strata(Pair.number), data = fartcd)

OR.CI <- cbind("OR" = exp(coef(fit.clr)), exp(confint(fit.clr)))

round(OR.CI, 3)

summary(fartcd$cat_PFDA)

summary(fit.clr) #results used in fart2021 dataframe

cdpfda = list(

lochhead2021 = data.frame(

stlab = c("Lochhead et al., 2021","",""),

labs = c("0.04-0.13 ng/ml","0.13-0.24 ng/ml","0.24-1.21 ng/ml"),

cutpoints = c(0.04, 0.13, 0.24),

RR = c(1.00, 0.59, 0.39),

lower = c(1.00, 0.29, 0.17),

upper= c(1.00, 1.20, 0.92)),

fart2021 = data.frame(

stlab = c("Fart et al., 2021",""),

labs = c("0.03-0.257 ng/mL", ">0.257 ng/mL"),

cutpoints = c(0, 1),

RR = c(1, 0.20),

lower = c(1, 0.044),

upper = c(1, 0.913))

)

cdpfda2 = lapply(cdpfda, function(df) {

cp = df$cutpoints

l = length(cp) + 1

cp[l] = 4 * cp[l-1] # assume max is 4*last cutpoint

df$mids = apply(rbind(cp[-l],cp[-1]),2,mean)

return(df)

})

cdpfda2$lochhead2021$mids[3] = mean(c(0.24,1.21))

cdpfda2$fart2021$mids[2] = mean(c(0.257,0.89))

trendp = function(datalist){

3

lapply(datalist, function(df) {

se1 = (log(df$upper)-log(df$RR))/qnorm(.975) # se of log RR for each dose category

se2 = (log(df$RR)-log(df$lower))/qnorm(.975)

se = (se1 + se2) / 2

scores = 0:(length(se)-1)

if(se[1] == 0) {

lm1 = lm(log(RR) ~ 0 + scores, weights = 1/se^2, data=df, subset=se>0)

lm2 = lm(log(RR) ~ 0 + mids, weights = 1 / se^2, data=df, subset=se>0)

p1 = summary(lm1)$coef[1,4]

p2 = summary(lm2)$coef[1,4]

slope = summary(lm2)$coef[1,1]

se = summary(lm2)$coef[1,2]

} else {

lm1 = lm(log(RR) ~ scores, weights = 1 / se^2, data=df)

lm2 = lm(log(RR) ~ mids, weights = 1 / se^2, data=df)

p1 = summary(lm1)$coef[2,4]

p2 = summary(lm2)$coef[2,4]

slope = summary(lm2)$coef[2,1]

se = summary(lm2)$coef[2,2]

}

return(c(p1,p2,slope,se))

})

}

(ktrend = trendp(cdpfda2))

fit.clr <- clogit(Case ~ PFDA + strata(Pair.number), data = fartcd)

ktrend$fart2021[3] <- coef(fit.clr)[1]

(coef(fit.clr))

ktrend$fart2021[4] <- (summary(fit.clr))$coef[3]

klogRR = c(ktrend$lochhead2021[3], ktrend$fart2021[3])

kse = c(ktrend$lochhead2021[4], ktrend$fart2021[4])

(m2 = metagen(klogRR,kse))

round(exp(c(m2$TE.fixed,m2$lower.fixed,m2$upper.fixed)),2)

round(exp(c(m2$TE.random,m2$lower.random,m2$upper.random)),2)

krows = unlist(lapply(cdpfda2,function(df) dim(df)[1]))

kRR = unlist(lapply(cdpfda2,function(df) df$RR))

klower = unlist(lapply(cdpfda2,function(df) df$lower))

kupper = unlist(lapply(cdpfda2,function(df) df$upper))

kstlabs = unlist(lapply(cdpfda2,function(df) df$stlab))

klabs = unlist(lapply(cdpfda2,function(df) df$labs))

klabels = cbind(c("Study", as.character(kstlabs)),

c("Exposure Category", as.character(klabs)))

png("cdpfda.png", width = 12, height = 9, units = 'in', res = 300)

ltg = "gray80"

forestplot(labeltext=klabels, graph.pos=3,

mean=c(NA, log(kRR)),

lower=c(NA, log(klower)),

upper=c(NA, log(kupper)),

title="Log Rate Ratio and 95% Confidence Interval by Study and Exposure Category for Crohn's Disease and PFDA",

hrzl_lines=list("2" = gpar(lwd=1, col=ltg),"5" = gpar(lwd=1, col=ltg), "7" = gpar(lwd=1, col=ltg)),

xticks=-2:2,

txt_gp=fpTxtGp(label=gpar(cex=1.25),

ticks=gpar(cex=1.1),

xlab=gpar(cex = 1.2),

title=gpar(cex = 1.2)),

col=fpColors(box="#1c61b6", lines="#1c61b6", zero = "tomato"),

zero=0, cex=0.9, lineheight = "auto", boxsize=0.3, colgap=unit(10,"mm"),

lwd.ci=2, ci.vertices=TRUE, ci.vertices.height = 0.2)

dev.off()
